# Three years of medication-use sequences in incident bipolar disorder in Sweden reveal divergent patterns in native-born and immigrant populations

**DOI:** 10.1038/s41398-025-03723-7

**Published:** 2026-01-14

**Authors:** Alexander Kautzky, Katalin Gémes, Bergný Ármannsdóttir, Ridwanul Amin, Aemal Akhtar, Johannes Lieslehto, Antti Tanskanen, Heidi Taipale, Ellenor Mittendorfer-Rutz

**Affiliations:** 1 Division of Insurance Medicine, Department of Clinical Neuroscience, Karolinska Institutet, Stockholm, Sweden; 2 Department of Psychiatry and Psychotherapy, Medical University of Vienna, Vienna, Austria; 3 Division of Infectious Diseases, Department of Medicine, Karolinska Institutet, Stockholm, Sweden; 4 Department of Forensic Psychiatry, University of Eastern Finland, Niuvanniemi Hospital, Kuopio, Finland; 5 School of Pharmacy, University of Eastern Finland, Kuopio, Finland

**Keywords:** Bipolar disorder, Clinical pharmacology

## Abstract

Guideline-conform treatment of mental disorders is compromised in immigrant populations, but longitudinal pharmacoepidemiologic patterns in bipolar disorder (BD) remain unknown. We aimed to close this knowledge gap by applying state sequence analysis (SSA) to comprehensively assess individual-level medication use. Psychopharmacological medication use was assessed among Swedish-born, second-generation, non-refugee and refugee first-generation immigrants with incident BD diagnosed in Sweden 2006–2015 (*n* = 24,578, 16–65 years). Three years of medication-use were conceptualized with SSA as consecutive sequences of three-month periods. Anticonvulsant mood-stabilizer, lithium and antipsychotic use was considered adequate treatment. Typologies were identified by clustering and associated with population groups and covariates applying multinomial logistic regression, yielding odds ratios (OR) for comparison to the majority typology as well as estimated probabilities for each typology. Immigrant populations discontinued medication within 6 months more frequently than Swedish-born (42.1–45.7% vs 36.8%). Transitions from periods lacking medication to adequate treatment showed low likelihood across population groups (8.9–10.1%). T*reatment failure* (48.3% of refugees, 32.3% of Swedish-born), representing lack of adequate and antidepressant medication, predominated among seven identified typologies. Compared to Swedish-born and *treatment failure*, adjusted OR for other typologies were lower for refugees (0.3–0.5) and other immigrant groups (0.5–0.8). Adjusting for covariates, highest probabilities for *treatment failure* were computed for non-refugee (44%) and refugee first-generation immigrants (51%), followed by individuals with low education level (42%) and psychiatric comorbidities (attention-deficit/hyperactivity disorder 38%, substance-use disorder 37%). In conclusion, immigrant groups, particularly refugees, with incident BD are less likely to receive adequate treatment, requiring special emphasis on guideline-conformance.

## Introduction

Bipolar disorder (BD) is a severe mood disorder with substantial disease burden and loss of healthy life expectancy [1]. Alternating depressive, manic, or mixed episodes complicate treatment that must be adaptive of episodes and acute and maintenance phases. Adequate treatment is further hindered by competing recommendations of established guidelines that include anticonvulsant mood-stabilizers, lithium, atypical antipsychotics and add-on antidepressants [2, 3]. Consequently, deviations from guideline recommendations such as late drug initiation, early discontinuation and antidepressant monotherapy are common, especially in ethnic minority and other socioeconomically disadvantaged groups [4].

In 2023, one in eight people globally and a quarter of the Swedish population had a migration history [5, 6]. Migration is oftentimes consequence but also cause of adversities that jeopardize mental health [7]. In addition to traumatic experiences before and during migration, in the new country of residence immigrants face new sociocultural environments, racial discrimination, and obstacles to labor market participation that will also affect second generations [8, 9]. Particularly refugees are known to be at increased risk of common as well as severe mental disorders [10, 11]. Results on prevalence of BD in migrant groups have been conflicting [12], likely owed to diagnostic challenges related to the episodic nature of BD [13]. Some studies suggested BD to be less common in first-generation immigrants but to increase in second generations [14].

Nevertheless, research addressing treatment of BD in immigrant groups is scarce. Among individuals living in Sweden with common mental disorders, lower probabilities of both initiation of [15] and adherence to [16] adequate medication were observed among immigrant groups compared to the host populations. Similar although less pronounced treatment gaps were found between refugees and the Swedish host population regarding first episode psychosis [17]. Regarding BD, use rates of medication targeting BD in the 5 years after incident diagnosis were significantly lower in both, first-generation immigrants and their descendants, compared to Swedish-born individuals [18]. All immigrant groups but especially refugees had low use of mood-stabilizers and high rates of lacking any treatment.

Nevertheless, longitudinal patterns of transitions between different medication classes and treatment discontinuation remain largely unknown in BD. Based on previous research, contributing effects of migration history, sociodemographic variables such as sex, age and educational level, but also individual medical history of comorbidities, must be considered. Sophisticated data mining approaches are required to overcome simplified outcomes such as initiation or discontinuation of specific medication types while also accounting for population groups and covariates [19, 20]. State sequence analysis was applied for investigating pharmacoepideiological patterns such as individual medication-use profiles in depression and psychosis [21–24]. Combined with other statistical tools such as clustering and multinomial regression, typologies representative of sequences shared between individuals with BD as well as associated covariate profiles can be identified.

Thus, we aim at three years of individual-level follow-up after incident diagnosis of BD to investigate sequences of treatment initiation, discontinuation and transitions between psychopharmacological classes, respectively among Swedish-born, refugee and non-refugee first-generation immigrants as well as second-generation immigrants. Further, we aim at identifying typologies of medication use that can be related to sociodemographic and clinical profiles representative of subgroups at risk of inadequate treatment.

## Methods

### Study cohort

Swedish national registers (as detailed in Supplementary Table 1) were linked by pseudonymized personal identification numbers. Individuals aged 16–65 who received a diagnosis of BD between 01/07/2006 and 31/12/2015, defined by codes F30 or F31 according to the tenth instalment of the international classification of diseases (ICD-10), were selected by secondary (inpatient and specialized outpatient) healthcare records or by sickness absence and disability pension spells due to these diagnoses. To select incident diagnosis, first we excluded individuals with any BD diagnosis during the three-years prior to inclusion. Second, we excluded individuals who used antipsychotics or mood-stabilizers from 15 up until 3 months before diagnosis. To maintain a sample primarily representative of BD, we further excluded individuals diagnosed in secondary healthcare with psychosis spectrum (ICD-10: F2) or dementia (ICD-10: F00-03, G30) within three years prior to BD or throughout the follow-up. Individuals were, therefore, required to have lived in Sweden continuously for these three years prior to BD diagnosis as well as throughout the complete follow-up or until death. Consequently, people who emigrated from Sweden throughout the follow-up were also excluded.

The project was approved by the Regional Ethical Review Board, Karolinska Institutet, Stockholm, Sweden (review number Dnr: 2007/762-31 and Dnr 2021-06441-02). For this register study without participant contact informed consent was not required in accordance with Swedish legislation.

### Population groups

Four population groups were defined by country of birth and refugee status. Countries of origin and residence duration in Sweden of immigrant groups are provided in Supplementary Table 2.

1. Swedish-born; subjects and both their parents born in Sweden (missing information of one parent was allowed).
2. Refugees: subjects who received refugee status by the Swedish migration agency.
3. Non-refugee immigrants: subjects without refugee status born in any country that was also registered as country of birth among the refugees group.
4. Second-generation immigrants: subjects born in Sweden with at least one parent having immigrated to Sweden from any country that was also registered as country of birth among the refugee group (missing information of one parent was allowed).

### Medication use & sequence states

PRE2DUP was used to track dispensing dates and model longitudinal medication use [25]. Based on sliding averages of dispensed daily dosages rather than prescriptions, PRE2DUP allows a realistic assessment of daily medication use that is sensitive to stockpiling, personal purchase patterns and hospitalization. Medication types were identified by Anatomical Therapeutic Chemical classification system (ATC) codes and grouped as (1) antidepressants, (2) anticonvulsant mood-stabilizers, (3) lithium, and (4) antipsychotics. Details on ATC codes and cumulative exposure days are provided in Supplementary Table 3.

Starting from incident BD diagnosis, twelve consecutive periods of three months (91 days) were defined, adding up to roughly 3 years (1092 days) of follow-up. For each period, daily use of the four medication groups was modelled to binary indicators dependent on the condition of usage on ≥ 50% of the days, thus allowing within-class switches. Inpatient days were subtracted from each period as medication received in hospital is not covered by PRE2DUP. Medication use was not assessed for periods with ≥ 50% of the days spent in hospital, in which case the following period was used as substitute. Subjects with inpatients stays spanning over ≥ 50% of the days of two or more consecutive periods were excluded. In case of death, a censoring state was assigned from the period of occurrence until end of follow-up. Subjects censored already from the first period were excluded from further analysis.

**Fig. 1:**
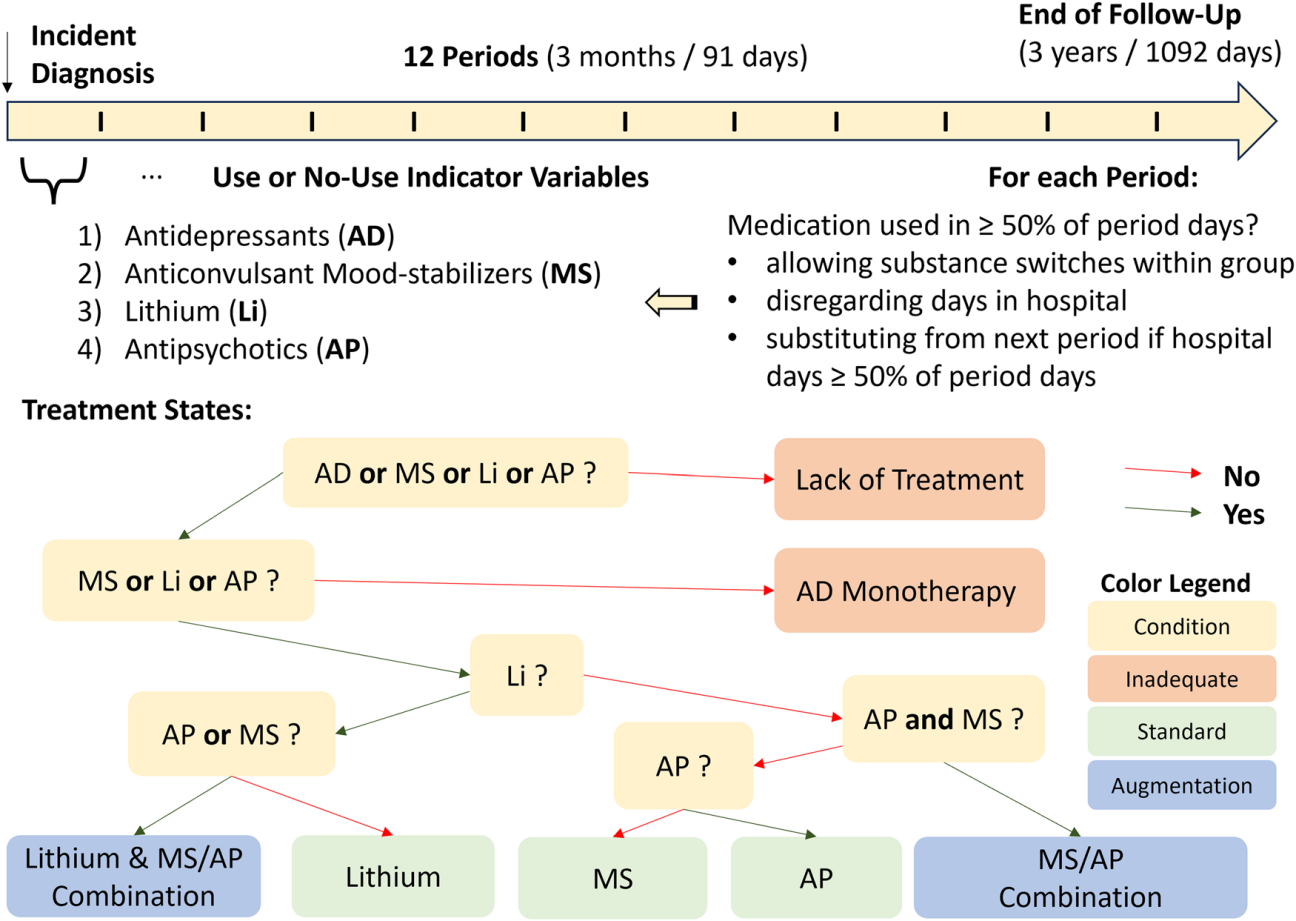
Schematic depiction of the study design and state definition for sequence analysis.

Based on the indicators of the four medication groups, and considering our previous observation of antidepressants used by the majority of individuals treated for incident BD in Sweden, seven mutually exclusive treatment states were defined and are presented as a decision tree in Fig. 1:

Inadequate Treatment States:

1. “Lack of Treatment”: all indicators negative.
2. “Antidepressant Monotherapy”: positive indicator for antidepressants, negative indicators for other medication groups.
  Standard Treatment States (concomitant antidepressants allowed):
3. “Mood-stabilizers”: positive indicator for anticonvulsant mood-stabilizers, negative indicators for antipsychotics and lithium.
4. “Antipsychotics”: positive indicator for antipsychotics, negative indicators for anticonvulsant mood-stabilizers and lithium.
5. “Lithium”: positive indicator for lithium, negative indicators for anticonvulsant mood-stabilizers and antipsychotics.
  Augmentation Therapy States (concomitant antidepressants allowed):
6. “Augmentation”: positive indicator for antipsychotics and anticonvulsant mood-stabilizers, negative indicators for lithium.
7. “Lithium with Augmentation”: positive indicator for lithium and at least one of anticonvulsant mood-stabilizers or antipsychotics.

### Covariates

Socio-demographic variables, including sex, age, educational level and family situation, were assessed in the year prior to diagnosis. Health-related variables, including history of sickness absence, disability pension and suicide attempt, and mental and somatic comorbidities diagnosed in secondary healthcare as main or side diagnosis defined by ICD-10 codes, were assessed for the three years prior to incident BD diagnosis. Supplementary Table 4 presents a detailed characterization of covariates.

### Statistics

#### Sequence analysis

First, the full spectrum and the most common sequences were identified for each population group, respectively. Further, the distribution of states across periods and transversal and longitudinal entropy, i.e., the distribution of states within each period and within sequences across the whole follow-up, were computed. Mean time spent within each state was computed per population group and differences tested by log-linear regression.

#### Treatment initiation and discontinuation

For analysis of treatment patterns regarding initiation and discontinuation, the sequence analysis design was simplified, dichotomizing the seven treatment states described above to (1) “Lack of Treatment” and (2) any of the remaining states, termed “Treatment”.

Treatment initiation was defined as the first transition from a state of “Lack of Treatment” to “Treatment”. Time to treatment initiation was defined as the number of periods from diagnosis until the respective event and results are presented descriptively by numbers and percentages among each population group. Early treatment was defined by initiation within 3 months after diagnosis, otherwise treatment was regarded delayed. Antidepressant monotherapy before diagnosis of BD that continued thereafter was also considered early treatment.

Patients who initiated treatment within a year after diagnosis were also assessed for discontinuation. Discontinuation was identified by the first transition from “Treatment” to “Lack of Treatment”, and time to discontinuation was defined as the number of periods until this event occurred. For easier interpretability, trajectories representing early and delayed initiation as well as time to discontinuation are presented descriptively for each population group.

#### Typologies

For identification of typologies, first a dissimilarity matrix of observed sequences was computed with the “optimal matching” method and applying a substitution-cost matrix sensitive to the observed transitions between states. We used hierarchical clustering with the “Ward” method and partitioning around medoids (PAM), as well as the combination of both. The optimal number of typologies was identified among a range of 2–15 clusters by optimization of average silhouette width (ASW; ranging from 0–1, thresholds of 0.3 and 0.5, respectively, indicating a reasonable and good fit), while considering the data, context and interpretability.

Next, multinomial logistic regression models were estimated to assess univariate associations between population-group and typologies. Results were compared to multivariate models adjusted for covariates. Fitted probabilities for each typology respective of population-group were computed, and respective of covariate factor levels after stratification by population-group. In addition, odds ratios (OR) with 95% confidence intervals (CI) for each typology were computed for immigrant population groups, relative to the majority population of Swedish-born and the largest sequence typology.

The packages “TraMineR” and “weightedCluster” for the statistical software R were used respectively for analysis and visualization of sequences of treatment states.

## Results

A total of 24,578 individuals were included, comprising 20,361 (82.8%) Swedish-born, 1867 (7.6%) second-generation immigrants, 1462 (5.9%) non-refugee immigrants, and 888 (3.6%) refugees. As detailed in Supplementary Table 4, psychiatric diagnoses received during 3 years before BD were common and rates comparable across population groups. Depression (F32-39) accounted for the majority of prior psychiatric diagnoses in all groups, however, antidepressant use within 3–15 months prior to BD diagnosis was less common in immigrant groups than Swedish-born. Further differences between groups included higher age, education level and rates of living with partners and/or children in first-generation immigrants compared to second-generation immigrants and Swedish-born.

### Sequence analysis

**Fig. 2:**
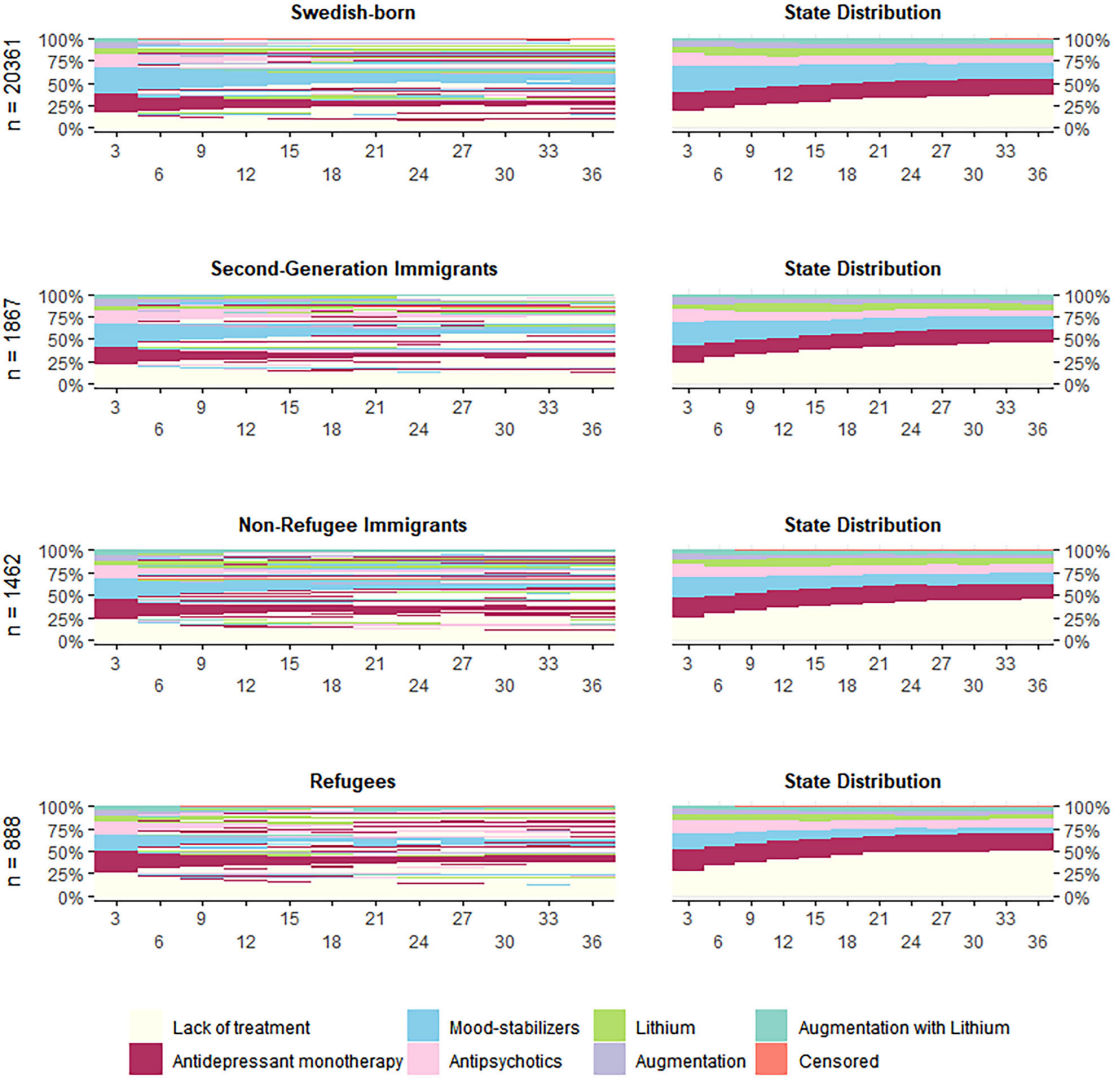
Sequence analysis of three years of medication-use in all individuals with incident diagnosis of bipolar disorder in Sweden 2006–2015, respectively for population groups defined by migration history. Observed sequences are paired with state distribution for each 3-month period, respectively for population-groups. Months are shown on the x-axis, proportions on the y-axis.

A total of 9723 different sequences of medication use were observed, displayed in Fig. 2. Similar sequence complexity and degrees of variation of states across periods were computed across population-groups (Supplementary Fig. 1).

“Lack of treatment” was continuously the most frequent state among refugees and non-refugee immigrants. In contrast, “mood-stabilizers” was the most frequent state observed in the first 3 months after diagnosis in second-generation immigrants and Swedish-born. Across follow-up, “lack of treatment” steadily increased and became the most frequent state in all groups but remained less common in Swedish-born (36.4%) compared to immigrant groups (45.1–50.6%) three years after diagnosis.

### Treatment initiation and discontinuation

Treatment initiation at some point across follow-up was seen in 91.5% of Swedish-born and 86.6–89.1% of immigrant groups (Table 1, section A). Among those with treatment, timely initiation within the first 3 months after diagnosis was common in all population groups with highest rates in Swedish-born and lowest in refugees.

**Table 1:** Three years of medication-use patterns in all individuals with incident diagnosis of bipolar disorder in Sweden 2006–2015, respectively for population groups defined by migration history.

	Swedish-born	Second-generation immigrants	First-generation immigrants	
			Non-refugees	Refugees
A) Treatment Initiation	*n* = 20361	*n* = 1867	*n* = 1462	*n* = 888
Yes	18640 (91.5%)	1633 (87.5%)	1302 (89.1%)	769 (86.6%)
No	1721 (8.5%)	234 (12.5%)	160 (10.9%)	119 (13.4%)
Time to Initiation	*n* = 18640	*n* = 1633	*n* = 1302	*n* = 769
Early, 0–3 months	16634 (89.2%)	1431 (87.6%)	1117 (85.8%)	641 (83.4%)
Delayed, 4–12 months	1357 (7.3%)	136 (8.3%)	124 (9.5%)	84 (10.9%)
Delayed, ≥12 months	649 (3.5%)	66 (4.0%)	61 (4.7%)	44 (5.7%)
Discontinuation^a^	*n* = 17991	*n* = 1567	*n* = 1241	*n* = 725
No	9937 (55.2%)	724 (46.2%)	562 (45.3%)	290 (40.0%)
Yes	8054 (44.8%)	843 (53.8%)	679 (54.7%)	435 (60.0%)
Time to Discontinuation^b^	*n* = 8054	*n* = 843	*n* = 679	*n* = 435
Within 6 Months	2963 (36.8%)	355 (42.1%)	288 (42.4%)	199 (45.7%)
Within 1 Year	1727 (21.4%)	156 (18.5%)	150 (22.1%)	99 (22.8%)
Within 2 Years	2320 (28.8%)	229 (27.2%)	169 (24.9%)	109 (25.1%)
After 2 Years	1044 (13.0%)	103 (12.2%)	72 (10.6%)	28 (6.4%)
B) Treatment Trajectory	*n* = 20361	*n* = 1867	*n* = 1462	*n* = 888
Early Start, no Discontinuation	9399 (46.2%)	683 (36.6%)	521 (35.6%)	272 (30.6%)
Early Start, Discontinuation	7235 (35.5%)	748 (40.1%)	596 (40.8%)	369 (41.6%)
Delayed Start, no Discontinuation	851 (4.2%)	68 (3.6%)	65 (4.4%)	34 (3.8%)
Delayed Start, Discontinuation	1155 (5.7%)	134 (7.2%)	120 (8.2%)	94 (10.6%)
Continuous Lack of Treatment	1721 (8.5%)	234 (12.5%)	160 (10.9%)	119 (13.4%)
C) Treatment Typology	*n* = 20361	*n* = 1867	*n* = 1462	*n* = 888
Treatment failure	6586 (32.3%)	762 (40.8%)	605 (41.4%)	429 (48.3%)
AD monotherapy	3851 (18.9%)	309 (16.6%)	275 (18.8%)	166 (18.7%)
Persistent Mood-stabilizers	4256 (20.9%)	306 (16.4%)	205 (14.0%)	80 (9.0%)
Persistant Antipsychotics	1838 (9.0%)	147 (7.9%)	131 (9.0%)	81 (9.1%)
Persistant Lithium	1725 (8.5%)	149 (8.0%)	116 (7.9%)	50 (5.6%)
Persistant Augmentation	1053 (5.2%)	92 (4.9%)	60 (4.1%)	33 (3.7%)
Persistant Augmentation with Lithium	1052 (5.2%)	102 (5.5%)	70 (4.8%)	49 (5.5%)

(A) Rates of treatment initiation and discontinuation with respective time until the event. (B) Trajectories of treatment initiation and discontinuation. (C) Typologies identified by clustering, representing predominant medication use throughout follow-up.

^a^Among subjects starting treatment within a year after diagnosis.

^b^Among subjects who discontinue treatment after having started within a year after diagnosis.

Discontinuation at any period across follow-up was particularly common in refugees (60.0%; vs 44.8% in Swedish-born). Among those with discontinuation, immigrant groups had higher rates of early discontinuation within 6 months after initiation compared to Swedish-born.

Synthesizing these results, early treatment initiation without discontinuation was the most frequent trajectory among Swedish-born (46.2%; vs 30.6–36.6% in immigrant groups; detailed in Table 1, section B). In contrast, early treatment initiation with discontinuation was the most frequent trajectory among immigrant groups (40.1–41.6%; vs 35.5% in Swedish-born). Continuous lack of treatment was rare in Swedish-born (8.5%) but more frequent in immigrant groups and particularly refugees (10.9–13.4%). For a graphical depiction of sequences of treatment initiation and discontinuation, please also see Supplementary Fig. 2.

### Mean time spent in states, transitions and sequence complexity

**Fig. 3:**
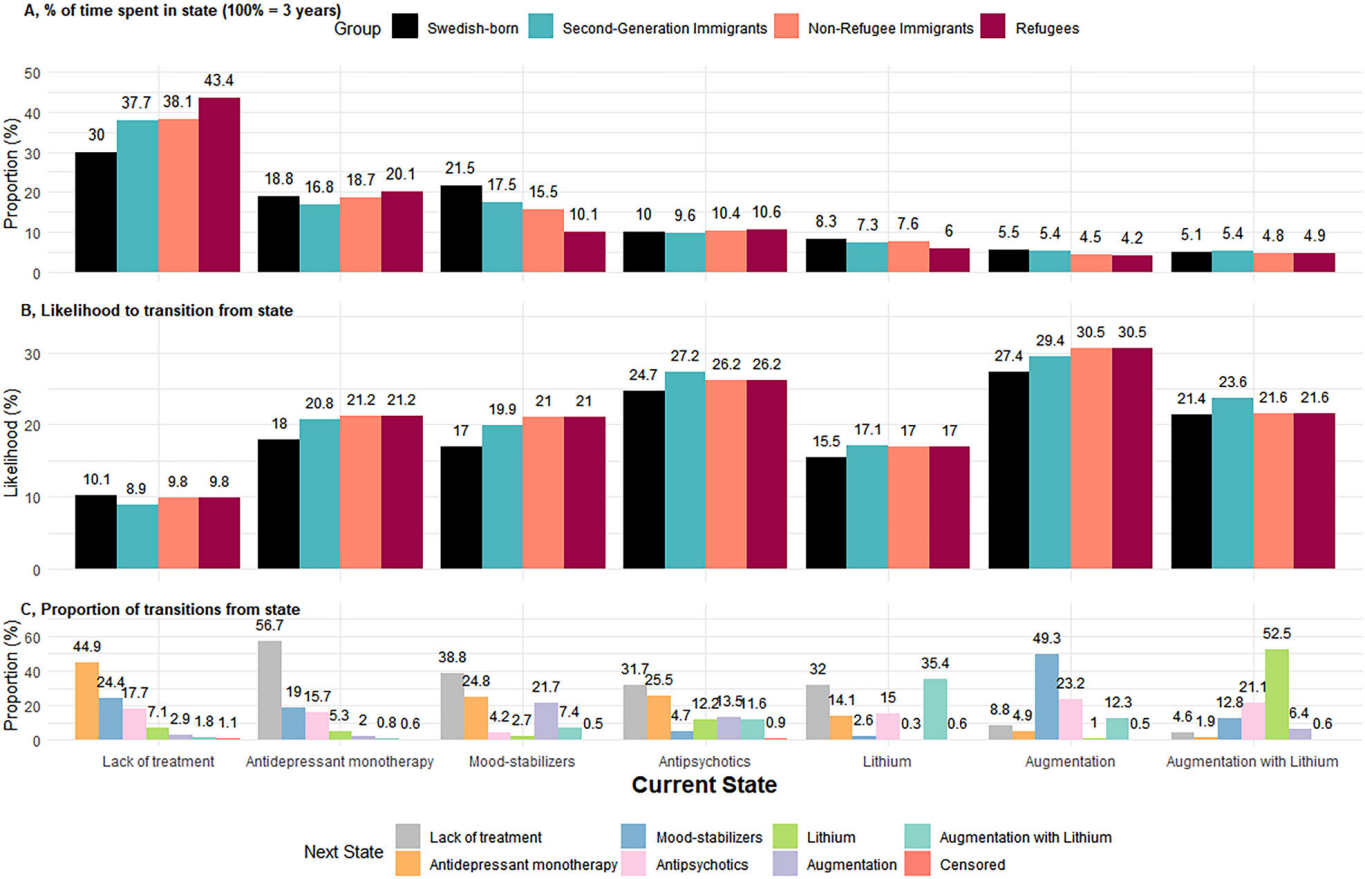
Evaluation of treatment states across a three-year follow-up of all individuals with incident diagnosis of bipolar disorder in Sweden 2006–2015, respectively for population groups defined by migration history. **A** Proportion of time spent in each state, respectively for population-groups. **B** Likelihood to transition from a specific state to any other state across each period, respectively for population-groups. **C** Proportion of transitions from each specific state to respective other states.

Among all population-groups, the largest proportion of the total follow-up time was spent in “lack of treatment” states (Fig. 3A). Nevertheless, large differences were observed between refugees (43.4%) and Swedish-born (30.0%). The next most common state was “mood-stabilizers” among Swedish-born (21.5%) and 2nd-generation immigrants (17.5%), but “antidepressant monotherapy” for non-refugee first-generation immigrants (18.8%) and refugees (20.1%). Refugees spent significantly less time in “mood-stabilizers” and more time in inadequate treatment states of either “antidepressant monotherapy” or “lack of treatment” than other groups. A complete list of contrasts between population groups is provided in Supplementary Table 5.

Regarding stability of states, likelihood of transitioning from “Lack of Treatment” to any treatment states was particularly low (8.9–10.2% across population groups). Transitions most commonly lead to the other inadequate treatment state, “antidepressant monotherapy” (44.9% of Transitions; Fig. 3B, C). The second most stable state was “lithium”, with 15.5–17.1% probabilities of transitioning. The largest proportion of these transitions represented augmentations with antipsychotics or anticonvulsant mood-stabilizers. Higher likelihoods of transitioning were estimated for states of “augmentation” and “antipsychotics”, however, mostly indicated adequate treatment mitigation as probabilities of transitions to inadequate treatment states were low (13.7% in “augmentation” and 6.5% in “lithium with augmentation”).

### Typologies of sequences of medication use

Cluster solutions with 5–7 typologies were indicated as optimal by both PAM and hierarchical clustering, detailed in Supplementary Table 6. Highest ASW of 0.51, indicating good fit, was achieved with PAM and 7 clusters. Supplementary Fig. 3 outlines the silhouettes of each cluster, while a confusion matrix of the different solutions is provided in Supplementary Table 7.

**Fig. 4:**
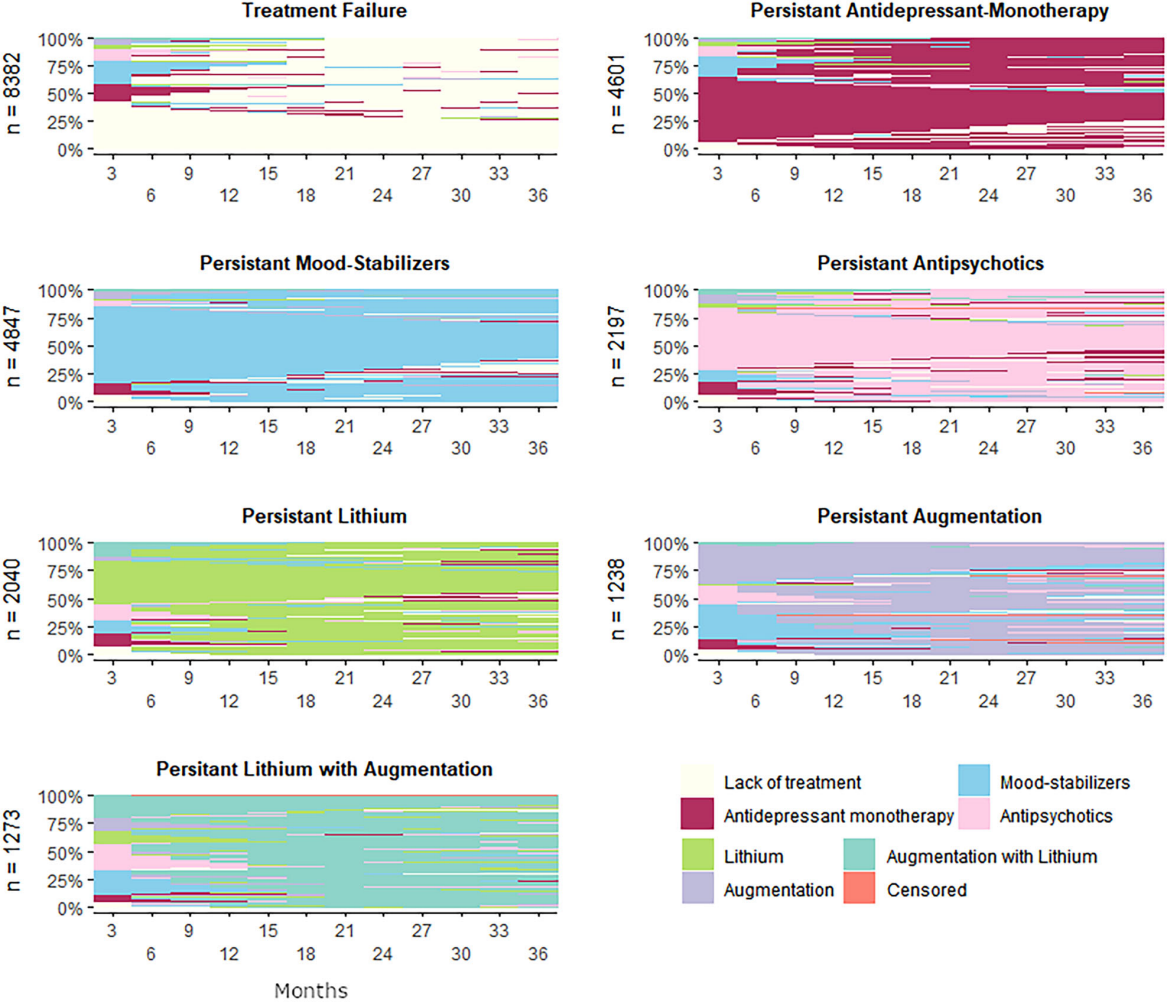
Observed sequences within each of the seven clusters according to partitioning around medoids. Cluster names are based on the representative sequence patterns. Sequences are ordered top to bottom by fit indices (average silhouette width), indicating stability of partitioning of each sequence within the respective cluster.

The spectra of sequences observed within the 7 typologies are displayed in Fig. 4 (for state distribution, see Supplementary Fig. 4). They represented respectively lack or discontinuation of treatment (termed *treatment failure*; 34.1% of individuals), predominant use of only antidepressants (termed *persistent antidepressant-monotherapy*, 18.7%), predominant use of either anticonvulsant mood-stabilizers (termed *persistent mood-stabilizers*, 19.7%), antipsychotics (termed *persistent antipsychotics*, 8.9%), or lithium (termed *persistent lithium*, 8.3%), as well as combinatory use of either anticonvulsant mood-stabilizers and antipsychotics (termed *persistent augmentation, 5.0%*), or lithium and either of anticonvulsant mood-stabilizers or antipsychotics (termed *persistent lithium with augmentation*, 5.2%).

As detailed in Table 1, section C, *treatment failure was* less frequent among Swedish-born than immigrant groups (32.3%, vs 40.8% in second-generation immigrants, 41.4% in non-refugee first-generation immigrants and 48.3% in refugees). Refugees showed lower rates of *persistent mood-stabilizers* (9%, vs 14.0–20.9%) and *persistent lithium* (5.6%, vs 7.9–8.5%). Little differences were observed for *persistent antidepressant-monotherapy*, *persistent antipsychotics* and both augmentation typologies. Taken together, typologies representing inadequate treatment deviating from guidelines were frequent, ranging from 51.2% in Swedish-born to 67.0% in refugees.

Among the typologies, *treatment failure* showed the highest proportion of subjects aged 16–35 years and with low education level but lowest proportions of subjects living with partner or children, as well as having any days of sickness absence in the year prior to diagnosis. A detailed characterization of clusters by socio-demographic covariates is provided in Supplementary Table 8.

### Multinomial regression

**Fig. 5:**
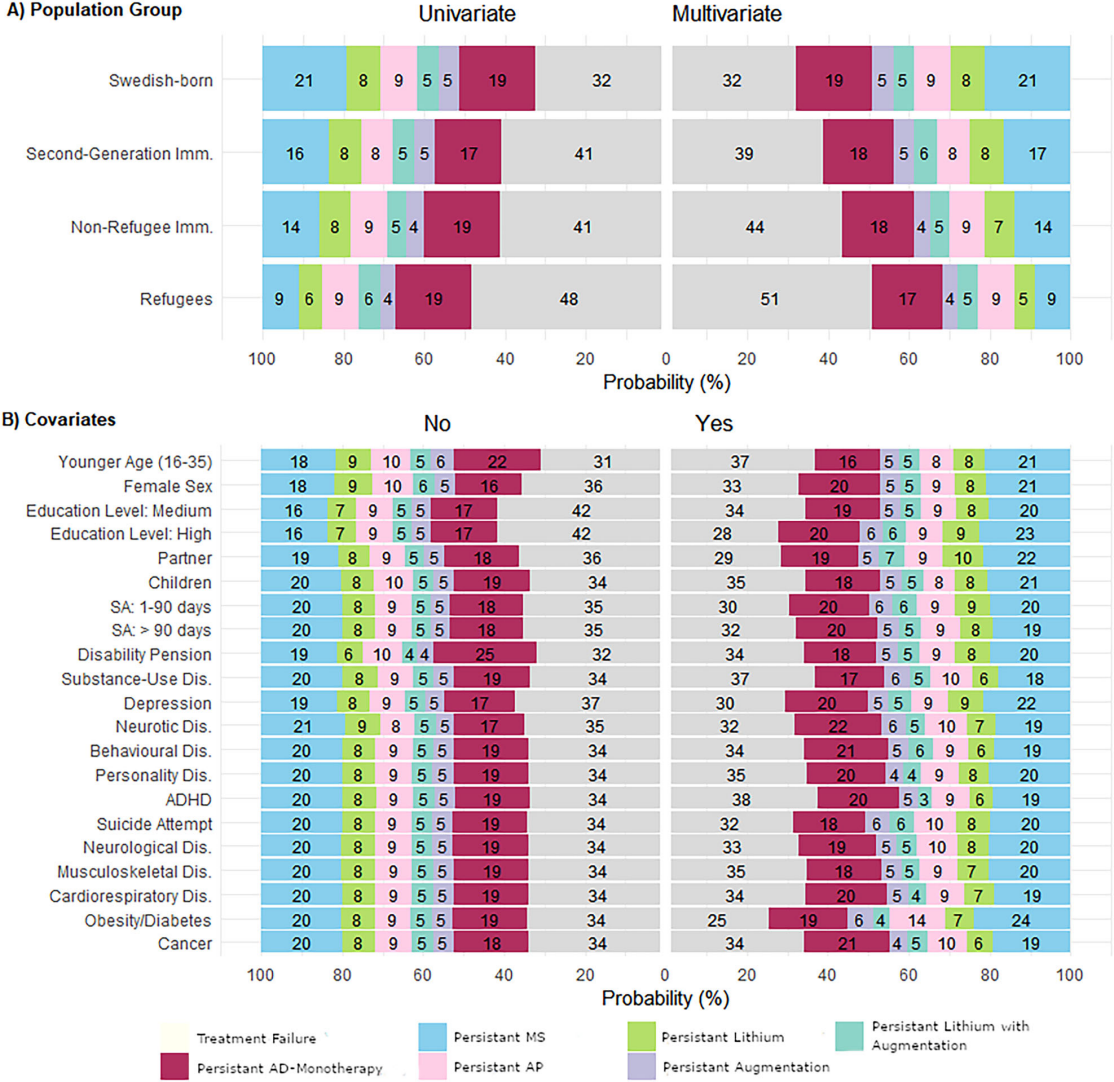
Multinomial regression results. **A** Estimated probabilities of cluster membership respectively for each population-group, respectively for univariate and multivariate analysis. **B** Effects of covariate levels on the estimated probabilities of cluster membership accords the whole study population.

Being the most prevalent cluster, *treatment failure* was used as reference for multinomial regression. Displayed in Fig. 5A, in the adjusted model refugees had the highest probability (51%, vs 32% in Swedish-born) of belonging to the *treatment failure* and the lowest probability of belonging to *persistent mood-stabilizers* (9%, vs 21% in Swedish-born) typology. The estimated probabilities of *persistent antidepressant-monotherapy* were similar between population-groups in the adjusted model (18–19%). As detailed in Supplementary Table 9, significantly lower odds of showing any typology other than *treatment failure* were computed for all immigrant groups but particularly refugees while adjusting for covariates.

Probabilities for each typology according to covariate levels are displayed in Fig. 5B. Across the whole population, highest estimated probabilities for *treatment failure* were observed for refugee (51%) and non-refugee (44%) first-generation immigrants and among those with low education levels (42%), comorbid attention-deficit/hyperactivity disorder (ADHD, 38%) and of substance-use disorder (37%) Highest estimates for *persistent antidepressant-monotherapy* were linked to disability pension (25%), comorbid neurotic disorders (22%) and older age of 36–65 years (22%). Highest probabilities for *persistent mood-stabilizers* were estimated for presence of obesity/diabetes (24%), while estimates for both *persistent mood-stabilizers* and *persistent lithium* were increased by high education level (23 and 9%), living with a partner (22 and 10%) and previous diagnosis of depression (22 and 9%).

Further stratified by population-group in Supplementary Fig. 5. Deviations from the general patterns were seen among refugees, who showed highest estimates for *treatment failure* in presence of comorbid substance-use disorder (66%) and young age of 16–35 years (62%). Contrary to other groups, education level hardly changed estimated probabilities for *treatment failure* in refugees (54–58%). Highest probabilities for *persistent antidepressant-monotherapy* were observed among comorbid ADHD (32%) and obesity/diabetes (29%).

## Discussion

Applying state sequence analysis to integrate Swedish registry data to three years of individual-level treatment patterns in subjects with incident BD revealed lower rates of initiation paired with higher rates of discontinuation of medication in immigrant groups and particularly refugees, compared to Swedish-born. Across all covariates analyzed, being first-generation non-refugee or refugee immigrant showed the highest risk for continued lack of adequate treatment. Immigrant populations used particularly less often mood-stabilizers including lithium, translating to increased frequencies of inadequate trials with antidepressant monotherapy among those who received treatment.

A majority of 72.2% of refugees and 81.7% of Swedish-born were using medication targeting BD in the first 3 month after diagnosis, comparative to reports from American prescription data of approximately 90% of individuals with BD being on pharmacological treatment within 6 months after incident diagnosis [26]. These numbers are not satisfactory considering that medication is recommended for any acute phase as well as in the maintenance phase of BD [27, 28]. Even more concerning are high discontinuation rates of up to 60% seen here in refugees, which are in line with previous reports of poor adherence in both short- and long-term treatment of BD in roughly half of cases [4]. These findings highlight the urgency of timely reach-out for treatment initiation as well as measures to uphold adherence across all population-groups, which currently are indicated to be unsuccessful in immigrant populations more often than in Swedish-born.

Regarding long-term patterns, the first treatment but also lack thereof was most likely to be retained or revisited throughout follow-up, as previously demonstrated for antipsychotic treatment in schizophrenia [21]. 89.2% of refugees and 92.5% of Swedish-born not treated in the first 3 months after diagnosis did not receive adequate medication at any point within the follow-up of 3 years. Consequently, lack of timely initiation or early discontinuation mostly persists throughout the three years after diagnosis, producing a typology of *treatment failure* observed in roughly half of refugees and a third of Swedish-born. The finding that the first medication is highly predictive of long-term treatment is also alarming regarding antidepressants. Roughly a quarter of all patients on medication, and more than third of first-generation immigrants, showed a pattern of *persistent antidepressant-monotherapy* throughout follow-up.

While antidepressants are suggested by most guidelines for BD as concomitant treatment options for bipolar depression, they are not recommended in the maintenance phase due to increased risk of mood switching while being of limited efficacy [3]. Despite growing evidence against the practice, antidepressant monotherapies are common and use only slightly declined over the past decade in many countries [29–31]. On an individual-level, we show here that treatment persistence was more likely in antidepressant monotherapy than any other medication. Treatment switches in patients on antidepressant monotherapy mostly indicated discontinuation, with a likelihood after any 3-month treatment period of 56.7%, while changes to more adequate options such as anticonvulsant mood-stabilizers (19%) or lithium (5.3%) were comparatively rare.

Taking a positive perspective, the general pattern to stick to medication administered in the acute phase of BD - assumed it was effective - agrees with guideline recommendations. Swedish-born as well as immigrant patients who received lithium as their first trial or switched to lithium early on, were most likely to transition between states of lithium monotherapy and augmentation throughout follow-up, while the risk of intermittent antidepressant monotherapies or complete discontinuation were comparatively low. Patients using anticonvulsant mood-stabilizers or antipsychotics early on showed similar patterns of frequent transitions to augmentation, albeit higher risk of discontinuation or switches to antidepressant monotherapy compared to lithium.

While this is the first study on longitudinal dispensation of different medication classes among migrant groups in BD, results can be contextualized with findings among other mental disorders. Lower and higher odds of respectively treatment initiation and discontinuation were found in immigrants compared to Finnish host-populations with depression as well as Swedish host-populations with common mental disorders [15, 16, 32]. Lower rates of initiation as well as lower adherence to antipsychotic medication were also found among migrant populations with non-affective psychosis compared to the host-populations of both Sweden and Finland [17, 33]. On a broader perspective, dispensing rates of most psychotropic medication classes were found to be reduced among migrant populations compared to the host-populations irrespective of diagnosis [34]. Treatment gaps in immigrant populations have been linked to socioeconomic factors such as education and income, structural deficits such as access to healthcare and the labour market, but also cultural factors among patients and stereotypes among healthcare providers [35–37]. Specifically, distrust following previous negative experiences with state structures, differences in mental health perception and a preference for alternative medication may contribute to higher risk of lowered adherence among immigrant groups. Racial stereotypes among healthcare providers and language barriers may contribute to difficulties forming a feasible therapeutic relationship. With regards to treatment with antidepressants and antipsychotics in migrant populations in Sweden, low education level and shorter duration of stay were previously associated with lower treatment initiation and a higher proportion of discontinuation, as well as labour market marginalization defined by long-term unemployment [38]. Specifically in BD, both low socioeconomic status and ethnic minority status were associated with poor treatment adherence [4]. While clinical severity was mostly similar across host- and immigrant populations, attitudes towards mental health and pharmacological treatment depend on education and cultural background and are known to predict treatment outcomes [39]. Here, low education level showed the strongest association with *treatment failure* when disregarding population group. However, higher probabilities of *treatment failure* were estimated for immigrant groups compared to Swedish-born also when accounting for education levels. In fact, the protective effect of high education levels regarding lack of treatment seen in Swedish-born was largely repealed in refugees, indicating that migration status constitutes a strong predictor of treatment course independently of education. On the other hand, high education level was more frequent in refugees than other populations, contradicting findings in the general population and indicating a selection bias. Refugees with low education level most likely face high risk of not getting in contact with the healthcare system and thus never receiving an adequate diagnosis of BD. Consequently, refugees with BD compared to the other population groups may include both clinically more severe cases with higher rates of involuntary contacts with the healthcare system but also the socioeconomically privileged who face less barriers to diagnosis and treatment relative to other refugees.

Reflecting on the results, state sequence analysis allows an explorative approach to pharmacoepidemiology useful for data reduction while maintaining individual-level information. Limitations include selection bias among the immigrant groups due to marginalization and limited access to healthcare but also based on misdiagnosis. Immigrant groups are long known to be at increased risk of being diagnosed with psychosis spectrum disorders rather than affective disorders, including BD [40]. While being beyond the scope of this study, diagnostic stability among patients with BD and psychosis spectrum disorders in relation to immigrant status may thus be an interesting focus for future research. Further limitations of the study are health care registers that lack symptom severity characteristics and do not allow direct assessment of clinical outcomes such as treatment success. Along these lines, higher rates of psychotic symptoms which may directly interfere with the ability to seek and adhere to treatment were reported among people with major depression and migration history [41]. Furthermore, psychosocial variables relevant to migrant groups such as language barriers and discrimination experiences cannot be captured by the Swedish national registers. On another note, non-pharmacological interventions could not be captured by this register-based analysis. Particularly migrant groups, who often face traumatic experiences before and after migration and are disadvantaged regarding mental health literacy, are known to benefit from psychoeducation and psychotherapeutic interventions. While going beyond the scope of this study, we would hypothesize that barriers similar to those affecting pharmacological treatment are relevant also for non-pharmacological interventions. Finally, some methodological limitations must be considered. We analysed dispensation of medication but cannot account for the potential that some individuals continuously filled prescriptions without taking the medication. Further, sequence states are defined arbitrarily and lack standardized statistical tests for interpretation. While the first limitation can be addressed by thoughtful study planning, the latter requires a thorough comparison of analytical approaches such as different clustering algorithms.

However, the register-based nature also brings along a major strength of the study as a broad range of socio-demographic and health-related characteristics of the complete Swedish population in secondary healthcare was included and followed up for three years.

## Conclusion

In summary, starting guideline-conform treatment as early as possible is key and especially so in immigrant groups. Both second- and first-generation immigrants, but particularly refugees, are at increased risk of never starting adequate treatment or early discontinuation. These gaps in medication use are the widest in anticonvulsant mood-stabilizers and lithium. The lower rates of adequate treatment and higher rate of antidepressant monotherapy may bring along unfavourable clinical outcomes such as more frequent or severe depressive and manic episodes as well as hospital readmissions, calling for further research aiming at BD in immigrant populations. Public health initiatives specifically targeting migrants with BD may be needed to address these inequalities.

## Supplementary information

Supplementary Section

## Data Availability

R code is available from the corresponding author upon scientific request. The used data cannot be made publicly available due to privacy regulations. According to the General Data Protection Regulation, the Swedish law SFS 2018:218, the Swedish Data Protection Act, the Swedish Ethical Review Act, and the Public Access to Information and Secrecy Act, these types of sensitive data can only be made available for specific purposes that meets the criteria for access to this type of sensitive and confidential data as determined by a legal review (contact email: imas-cns@ki.se).
