# Supplementary material for: Three years of medication-use sequences in incident bipolar disorder in Sweden reveal divergent patterns in native-born and immigrant populations
Source: Transl Psychiatry. 2026 Jan 14;16:38. doi: 10.1038/s41398-025-03723-7 (PMC12824395; doi:10.1038/s41398-025-03723-7)
Supplement: Supplementary file 1 — Supplementary Section [file 41398_2025_3723_MOESM1_ESM.docx]

| **Register** | **Type of Information** | **Reference** |
| --- | --- | --- |
| Longitudinal Database  for Integration Studies (STATIV) | refugee status and migration background for definition of 1^st^-generation immigrant and refugee groups | (Statistics Sweden, 2018) |
| Swedish Multi-generation Register | parental countries of birth for definition of the Swedish-born and 2^nd^-generation immigrant groups | (Ekbom, 2011) |
| National Patient Register (NPR) | dates of visits and main- and secondary-diagnoses from secondary healthcare in Sweden (specialized outpatient visits and inpatient stays) | (Ludvigsson *et al.*, 2011) |
| Prescribed Drug Register (PDR) | substances with dosages und quantities dispensed in Swedish pharmacies by Anatomical Therapeutic Chemical (ATC) codes | (Wettermark *et al.*, 2007) |
| Longitudinal Integration Database for Health Insurance and Labor Market Studies (LISA) | sociodemographic variables such as sex, education, living situation, place of residence, country of birth | (Ludvigsson *et al.*, 2019) |
| Total Population Register | emigration from Sweden, assessed annually at the end of each year | (Ludvigsson *et al.*, 2016) |
| Micro-Data for Analyses of Social Insurance (MIDAS) | dates and diagnoses regarding sickness absence and disability pension | (Försäkringskassan, 2018) |
| Cause of Death Register | date of death | - (Brooke *et al.*, 2017) |

**Supplementary Table 1.** Swedish national registries used for linkage of pseudonymized data on population-level. All registers are linked by the unique personal identification number attributed to every person living in Sweden.

|  | **2nd-generation immigrants** | | **1st-generation immigrants** | |
| --- | --- | --- | --- | --- |
| **Country** | **Mother (n=1867** | **Father (n=1867)** | **Non-refugee (n=1462)** | **Refugees (n=888)** |
| Sweden | 844 (44.4%) | 402 (21.1%) | 0% | 0% |
| Nordic Countries | 99 (5.2%) | 28 (1.5%) | 0% | 0% |
| Unknown | 7 (0.4%) | 25 (1.3%) | 0% | 0% |
| Iran | 40 (2.1%) | 83 (4.4%) | 88 (6.0%) | 138 (15.7%) |
| Bosnia and Herzegovina | 9 (0.5%) | 13 (0.7%) | 12 (0.8%) | 123 (14.0%) |
| Iraq | 12 (0.6%) | 26 (1.4%) | 61 (4.1%) | 91 (10.3%) |
| Former Yugoslavia | 112 (5.9%) | 178 (9.4%) | 73 (5.0%) | 75 (8.5%) |
| Chile | 40 (2.1%) | 69 (3.6%) | 101 (6.9%) | 51 (5.8%) |
| Turkiye | 44 (2.3%) | 71 (3.7%) | 50 (3.4%) | 26 (3.0%) |
| Poland | 131 (6.9%) | 69 (3.6%) | 177 (12.0%) | 25 (2.8%) |
| Russia | 1 (0.1%) | / | 28 (1.9%) | 23 (2.6%) |
| Libanon | 12 (0.6%) | 23 (1.2%) | 30 (2.0%) | 17 (1.9%) |
| Somalia | / | / | 10 (0.7%) | 17 (1.9%) |
| Syria | 16 (0.8%) | 15 (0.8%) | 19 (1.3%) | 16 (1.8%) |
| USA | 29 (1.5%) | 52 (2.7%) | 63 (4.3%) | 15 (1.7%) |
| Peru | / | / | 10 (0.7%) | 13 (1.5%) |
| Brasil | / | / | 19 (1.3%) | 13 (1.5%) |
| Afghanistan | / | / | / | 11 (1.3%) |
| UK | / | 61 (3.2%) | 67 (4.6%) | 11 (1.3%) |
| Colombia | / | / | 58 (3.9%) | / |
| Germany | 114 (6.0%) | 175 (9.2%) | 57 (3.9%) | / |
| India | / | 12 (0.6%) | 54 (3.7%) | / |
| Thailand | 18 (0.9%) | / | 35 (2.4%) | / |
| Hungary | 29 (1.5%) | 64 (3.4%) | 33 (2.2%) | / |
| Romania | / | 13 (0.7%) | 32 (2.2%) | / |
| Sri Lanka | / | / | 31 (2.1%) | / |
| Greece | 14 (0.7%) | 39 (2.0%) | 25 (1.7%) | / |
| Etiopia | / | 10 (0.5%) | 17 (1.2%) | / |
| Italy | / | 47 (2.5%) | 15 (1.0%) | / |
| Marocco | 15 (0.8%) | 28 (1.5%) | 15 (1.0%) | / |
| Netherlands | 14 (0.7%) | 19 (1.0%) | 15 (1.0%) | / |
| Phillipines | 13 (0.7%) | / | 14 (1.0%) | / |
| France | 14 (0.7%) | 15 (0.8%) | 13 (0.9%) | / |
| Spain | / | 34 (1.8%) | 13 (0.9%) | / |
| Argentina | / | / | 13 (0.9%) | / |
| USSR | 18 (0.9%) | 17 (0.9%) | 12 (0.8%) | / |
| Estonia | 28 (1.5%) | 35 (1.8%) | 12 (0.8%) | / |
| **Duration of Residence** |  |  |  |  |
| 3-10 yrs | n.a. | n.a. | 354 (24.0%) | 227 (25.8%) |
| 11-20 yrs | n.a. | n.a. | 360 (24.5%) | 450 (51.1%) |
| >20 yrs | n.a. | n.a. | 758 (51.5%) | 203 (23.1%) |

**Supplementary Table 2.** Countries of birth and duration of residence in Sweden, respectively for non-refugee and refugee 1^st^-generation immigrants, and of mothers and fathers of 2^nd^-generation immigrants. In case of fewer than 10 individuals, counts are censored with “/” and countries censored for both 1^st^-generation immigrant groups are not listed. A total of 24.6% of refugee and 13.4% of non-refugee first-generation immigrants were born in these countries.

| **Medication** | **ATC Code** | **Exposure Days (mean + SD)** |
| --- | --- | --- |
| **Antidepressants** | **N06A** | 638.4 ± 549.7 |
| SSRI | -B | 383.9 ± 484.2 |
| SNRI | -X16, -X17, -X21 | 157.7 ± 356.9 |
| Mirtazapine | -X11 | 77.3 ± 238.1 |
| Bupropion | -X12 | 52.9 ± 187.4 |
| Other Antidepressants | Remaining Codes of N06A | 61.8 ± 216.0 |
| **Anticonvulsant Mood-Stabilizers** | **N03AG01, N03AX09, N03AF01 & -F02** | 360.4 ± 461.7 |
| Valproate | N03AG01 | 62.1 ± 211.2 |
| Lamotrigine | N03AX09 | 300.1 ± 443.3 |
| Carbamazepine, Oxcarbazepine | N03AF01 & -F02 | 5.9 ± 61.6 |
| **Lithium** | **N05AN01** | 183.4 ± 370.9 |
| **Antipsychotics** | **N05A,** excluding -N01 | 230.3 ± 370.1 |
| Quetiapine | -H04 | 140.8 ± 305.2 |
| Olanzapine | -H03 | 46.4 ± 163.1 |
| Aripiprazole | -X12 | 27.0 ± 126.6 |
| Risperidone, Paliperidone | -X08 & -X13 | 10.92 ± 81.6 |
| Other Antipsychotics | Remaining Codes of N05A | 23.3 ± 105.3 |

Abbreviations: SSRI = selective serotonin-reuptake inhibitors, SNRI = serotonin-noradrenaline-reuptake inhibitors

**Supplementary Table 3.** Medication classes and respective subclasses/agents, listed by ATC codes. Classes/agents that were used for definition of treatment states are highlighted in bold. Mean days with standard deviations of exposure across the follow‑up of three years are provided for each medication class/agent.

| **Characteristics**  (at year before diagnosis) | **Swedish-born** | **2^nd^-generation immigrants** | **1^st^-generation immigrants** | |  |
| --- | --- | --- | --- | --- | --- |
|  |  |  | **Non-refugees** | **Refugees** |  |
|  | 20361 (82.8%) | 1867 (7.6%) | 1462 (5.9%) | 888 (3.6%) |  |
| **Age (years)** |  |  |  |  |  |
| Mean (SD) | 36.0 (±12.8) | 31.5 (±11.5) | 38.8 (±12.0) | 37.9 (±11.2) |  |
| 16 - 35 | 10725 (52.7%) | 1277 (68.4%) | 628 (43.0%) | 390 (43.9%) |  |
| 36 - 65 | 9636 (47.3%) | 590 (31.6%) | 834 (57.0%) | 498 (56.1%) |  |
| **Sex** |  |  |  |  |  |
| Women | 12941 (63.6%) | 1194 (64.0%) | 951 (65.0%) | 545 (61.4%) |  |
| Men | 7420 (36.4%) | 673 (36.0%) | 511 (35.0%) | 343 (38.6%) |  |
| **Education (years)** | | |  |  |  |
| Basic (0-9) | 4594 (22.6%) | 485 (26.0%) | 296 (20.2%) | 214 (24.1%) |  |
| Secondary (10-12) | 9961 (48.9%) | 882 (47.2%) | 653 (44.7%) | 366 (41.2%) |  |
| Higher (>12) | 5806 (28.5%) | 500 (26.8%) | 513 (35.1%) | 308 (34.7%) |  |
| **Living Situation** | | |  |  |  |
| Alone | 12463 (61.2%) | 1343 (71.9%) | 772 (52.8%) | 450 (50.7%) |  |
| with Children | 1701 (8.4%) | 87 (4.7%) | 177 (12.1%) | 92 (10.4%) |  |
| with Partner | 4202 (20.6%) | 287 (15.4%) | 366 (25.0%) | 242 (27.3%) |  |
| with Partner & Children | 1995 (9.8%) | 150 (8.0%) | 147 (10.1%) | 104 (11.7%) |  |
| **Sickness Absence** | | | | |  |
| None | 12852 (63.1%) | 1303 (69.8%) | 965 (66.0%) | 562 (63.3%) |  |
| 1-90 days | 3334 (16.4%) | 254 (13.6%) | 210 (14.4%) | 133 (15.0%) |  |
| >90 days | 4175 (20.5%) | 310 (16.6%) | 287 (19.6%) | 193 (21.7%) |  |
| **Disability Pension** | | | |  |  |
| No | 17870 (87.8%) | 1705 (91.3%) | 1256 (85.9%) | 801 (90.2%) |  |
| Yes | 2491 (12.2%) | 162 (8.7%) | 206 (14.1%) | 87 (9.8%) |  |
| **History of Suicide Attempt** ^a^ | | | | |  |
| No | 18948 (93.1%) | 1741 (93.3%) | 1360 (93.0%) | 840 (94.6%) |  |
| Yes | 1413 (6.9%) | 126 (6.7%) | 102 (7.0%) | 48 (5.4%) |  |
| **Antidepressant Use** ^b^ | | | | |  |
| No | 10747 (52.8%) | 1183 (63.4%) | 927 (63.4) | 570 (64.2%) |  |
| Yes | 9614 (47.2%) | 684 (36.6%) | 535 (36.6%) | 318 (35.8%) |  |
| **Psychiatric Comorbidities** ^a^ | | | | |  |
| None | 7748 (38.1%) | 679 (36.4%) | 604 (41.3%) | 348 (39.2%) |  |
| Depression (F32-39) | 8320 (40.9%) | 754 (40.4%) | 576 (39.4%) | 386 (43.5%) |  |
| Neurotic Disorder (F40-49) | 7098 (34.9%) | 673 (36.0%) | 470 (32.1%) | 334 (37.6%) |  |
| Substance Use (F10-19) | 2605 (12.8%) | 208 (11.1%) | 151 (10.3%) | 73 (8.2%) |  |
| ADHD (F90) | 1562 (7.7%) | 154 (8.2%) | 70 (4.8%) | 36 (4.1%) |  |
| Personality Disorder (F60-69) | 1222 (6.0%) | 125 (6.7%) | 99 (6.8%) | 43 (4.8%) |  |
| Behavioral Disorder (F50-59) | 959 (4.7%) | 108 (5.8%) | 56 (3.8%) | 19 (2.1%) |  |
| **Somatic Comorbidities** ^a^ | | | | |  |
| None |  |  |  |  |  |
| Muscolosceletal Disorder | 3184 (15.6%) | 258 (13.8%) | 294 (20.1%) | 165 (18.6%) |  |
| Neurolgical Disorder | 1599 (7.9%) | 149 (8.0%) | 126 (8.6%) | 75 (8.4%) |  |
| Cancer | 1296 (6.4%) | 100 (5.4%) | 121 (8.3%) | 54 (6.1%) |  |
| Cardiorespiratory Disorder | 1338 (6.6%) | 78 (4.2%) | 112 (7.7%) | 55 (6.2%) |  |
| Obesity & Diabetes | 621 (3.1%) | 54 (2.9%) | 55 (3.8%) | 32 (3.6%) |  |

**Supplementary Table 4.** Population-based cohort of all individuals with incident BD living in Sweden for three years before as well as after diagnosis. Characteristics are presented by population group. If not stated otherwise, variables were based on the year prior to incident diagnosis of BD.

| **Treatment State** | **Contrast** | **ß** | **SE** | **z.ratio** | **corr-p** |
| --- | --- | --- | --- | --- | --- |
| Anticonvulsant Mood-Stabilizers | Swedish-born - 2^nd^-Generation Im. | 0.204 | 0.017 | 12.35 | <0.001 |
|  | Swedish-born - Non-Refugee Im. | 0.322 | 0.020 | 16.41 | <0.001 |
|  | Swedish-born - Refugees | 0.751 | 0.031 | 24.42 | <0.001 |
|  | 2^nd^-Generation Im. - Non-Refugee Im. | 0.118 | 0.025 | 4.72 | <0.001 |
|  | 2^nd^-Generation Im. - Refugees | 0.547 | 0.034 | 15.90 | <0.001 |
|  | Non-Refugee Im. - Refugees | 0.429 | 0.036 | 11.93 | <0.001 |
| Antidepressant-Monotherapy | Swedish-born - 2^nd^-Generation Im. | 0.115 | 0.017 | 6.78 | <0.001 |
|  | Swedish-born - Non-Refugee Im. | 0.009 | 0.018 | 0.52 | n.s. |
|  | Swedish-born - Refugees | -0.064 | 0.022 | -2.91 | n.s. |
|  | 2^nd^-Generation Im. - Non-Refugee Im. | -0.106 | 0.024 | -4.42 | <0.001 |
|  | 2^nd^-Generation Im. - Refugees | -0.179 | 0.027 | -6.63 | <0.001 |
|  | Non-Refugee Im. - Refuge Es | -0.074 | 0.028 | -2.65 | n.s. |
| Antipsychotics | Swedish-born - 2^nd^-Generation Im. | 0.039 | 0.022 | 1.72 | n.s. |
|  | Swedish-born - Non-Refugee Im. | -0.039 | 0.024 | -1.61 | n.s. |
|  | Swedish-born - Refugees | -0.052 | 0.030 | -1.69 | n.s. |
|  | 2^nd^-Generation Im. - Non-Refugee Im. | -0.078 | 0.032 | -2.45 | n.s. |
|  | 2^nd^-Generation Im. - Refugees | -0.090 | 0.037 | -2.46 | n.s. |
|  | Non-Refugee Im. - Refugees | -0.013 | 0.038 | -0.33 | n.s. |
| Augmentation | Swedish-born - 2^nd^-Generation Im. | 0.022 | 0.030 | 0.72 | n.s. |
|  | Swedish-born - Non-Refugee Im. | 0.205 | 0.037 | 5.60 | <0.001 |
|  | Swedish-born - Refugees | 0.274 | 0.048 | 5.69 | <0.001 |
|  | 2^nd^-Generation Im. - Non-Refugee Im. | 0.184 | 0.046 | 4.01 | <0.001 |
|  | 2^nd^-Generation Im. - Refugees | 0.252 | 0.055 | 4.55 | <0.001 |
|  | Non-Refugee Im. - Refugees | 0.068 | 0.059 | 1.15 | n.s. |
| Lack of Treatment | Swedish-born - 2^nd^-Generation Im. | -0.229 | 0.011 | -19.95 | <0.001 |
|  | Swedish-born - Non-Refugee Im. | -0.239 | 0.013 | -18.71 | <0.001 |
|  | Swedish-born - Refugees | -0.369 | 0.015 | -24.37 | <0.001 |
|  | 2^nd^-Generation Im. - Non-Refugee Im. | -0.010 | 0.016 | -0.60 | n.s. |
|  | 2^nd^-Generation Im. - Refugees | -0.140 | 0.018 | -7.67 | <0.001 |
|  | Non-Refugee Im. - Refugees | -0.130 | 0.019 | -6.82 | <0.001 |
| Lithium | Swedish-born - 2^nd^-Generation Im. | 0.128 | 0.026 | 4.99 | <0.001 |
|  | Swedish-born - Non-Refugee Im. | 0.098 | 0.028 | 3.47 | n.s. |
|  | Swedish-born - Refugees | 0.331 | 0.040 | 8.24 | <0.001 |
|  | 2^nd^-Generation Im. - Non-Refugee Im. | -0.030 | 0.037 | -0.80 | n.s. |
|  | 2^nd^-Generation Im. - Refugees | 0.203 | 0.047 | 4.36 | <0.001 |
|  | Non-Refugee Im. - Refugees | 0.233 | 0.048 | 4.84 | <0.001 |
| Lithium  Augmentation | Swedish-born - 2^nd^-Generation Im. | -0.059 | 0.030 | -1.96 | n.s. |
|  | Swedish-born - Non-Refugee Im. | 0.060 | 0.036 | 1.69 | n.s. |
|  | Swedish-born - Refugees | 0.039 | 0.045 | 0.88 | n.s. |
|  | 2^nd^-Generation Im. - Non-Refugee Im. | 0.120 | 0.045 | 2.66 | n.s. |
|  | 2^nd^-Generation Im. - Refugees | 0.099 | 0.053 | 1.88 | n.s. |
|  | Non-Refugee Im. - Refugees | -0.021 | 0.056 | -0.38 | n.s. |

**Supplementary Table 5.** Log-linear regression results for mean times spent in each state across the three-year follow up. Contrasts are corrected by the Tukey method. Abbreviations: ß = regression coefficient, SE = standard error, n.s. = not significant

|  | Optimal  Nr. Clusters | Optimal Parameter | 2^nd^ Best Nr. Clusters | 2^nd^ Best Parameter | 3^rd^ Best  Nr. Clusters | 3^rd^ Best Parameter |
| --- | --- | --- | --- | --- | --- | --- |
|  | **Partitioning around Medoids (PAM)** | | | | |  |
| PBC | 7 | 0.82 | 6 | 0.8 | 5 | 0.79 |
| HG | 7 | 0.97 | 6 | 0.95 | 8 | 0.94 |
| HGSD | 7 | 0.97 | 6 | 0.95 | 8 | 0.94 |
| **ASW** | 7 | 0.51 | 6 | 0.49 | 5 | 0.48 |
| CH | 5 | 5760 | 6 | 5366 | 7 | 5099 |
| CHsq | 7 | 13864 | 6 | 13229 | 5 | 13020 |
| HC | 7 | 0.03 | 6 | 0.04 | 5 | 0.05 |
|  |  | **Hierarchical Clustering (Ward method)** | | | | |
| PBC | 8 | 0.71 | 5 | 0.69 | 7 | 0.69 |
| HG | 8 | 0.88 | 7 | 0.85 | 6 | 0.83 |
| HGSD | 8 | 0.88 | 7 | 0.85 | 6 | 0.83 |
| **ASW** | 5 | 0.4 | 8 | 0.4 | 7 | 0.39 |
| CH | 2 | 5161 | 4 | 5077 | 3 | 5020 |
| CHsq | 5 | 9565 | 4 | 9336 | 8 | 9039 |
| HC | 8 | 0.72 | 7 | 0.68 | 6 | 0.65 |
|  |  | **PAM starting on Hierarchical Clustering** | | | | |
| PBC | 7 | 0.82 | 6 | 0.81 | 5 | 0.79 |
| HG | 7 | 0.97 | 6 | 0.95 | 8 | 0.94 |
| HGSD | 7 | 0.97 | 6 | 0.95 | 8 | 0.94 |
| **ASW** | 7 | 0.51 | 6 | 0.5 | 5 | 0.48 |
| CH | 5 | 5753 | 6 | 5361 | 7 | 5095 |
| CHsq | 7 | 13847 | 6 | 13221 | 5 | 13011 |
| HC | 7 | 0.03 | 6 | 0.04 | 5 | 0.05 |

**Supplementary Table 6.** Top 3 best performing solutions for cluster solutions ranging between 1 and 12, produced by the PAM algorithm and hierarchical clustering. Average silhouette width (ASW) was considered the main decision criterion, giving optimal fits for 5-7 clusters. Abbreviations: PBC = Point Biserial Correlation, HG = Hubert's Gamma, HGSD = Hubert's Gamma (Somers'D), CH = Calinski-Harabasz index, CHsq = Calinski-Harabasz index on squared distances, HC = Hubert's C coefficient.

| **PAM -** | **PAM- 7 Clusters** | | | | | | |
| --- | --- | --- | --- | --- | --- | --- | --- |
| **5 Clusters** | TF | AD | MS | AP | LI | AUG | LI AUG |
| TF | **99.0** | 0 | 0 | 0 | 0 | 0.3 | 0.7 |
| AD | 0 | **98.9** | 0 | 0.2 | 0 | 0.7 | 0.2 |
| MS | 0 | 0 | **90.3** | 0 | 0 | 7.9 | 1.8 |
| AP / AUG | 0.6 | 0.4 | 0.6 | **71.7** | 0.1 | **22.0** | 4.6 |
| LI / LI AUG | 0.3 | 0.2 | 0.5 | 0.8 | **64.3** | 2.2 | **31.7** |
| **6 Clusters** |  |  |  |  |  |  |  |
| TF | **99.7** | 0 | 0 | 0 | 0 | 0 | 0.3 |
| AD | 0 | **99.8** | 0 | 0 | 0 | 0 | 0.2 |
| MS | 0 | 0 | **98.4** | 0 | 0 | 0 | 1.6 |
| AP | 0 | 0 | 0 | **95.2** | 0 | 0 | 4.7 |
| LI / LI AUG | 0.6 | 0.2 | 0.3 | 0.6 | **66.7** | 0.1 | **31.6** |
| AUG | 0 | 0 | 2.2 | 0 | 0 | **91.4** | 6.4 |
| **HC - 7** | TF | AD | MS | AP | LI | AUG | LI AUG |
| TF | **93.1** | 4.9 | 0 | 0.4 | 1.4 | 0.1 | 0.1 |
| AD | 6.2 | **82.0** | 4.1 | 3.8 | 2.0 | 1.1 | 0.9 |
| MS | 0 | 0 | **100** | 0 | 0 | 0 | 0 |
| AP | 4.9 | 0.2 | 2.1 | **87.7** | 2.3 | 1.6 | 1.2 |
| LI | 5.2 | 4.1 | 0.9 | 1.8 | **83.9** | 1.0 | 3.2 |
| MS / AUG / TF | 21.2 | 1.9 | 44.8 | 1.5 | 1.1 | 26.7 | 2.9 |
| LI AUG | 3.8 | 0.6 | 0.7 | 1.3 | 9.9 | 0.3 | **83.4** |

**Supplementary Table 7.** Confusion matrix for cluster solutions with 7 and respectively 6 and 5 clusters produced by the PAM algorithm, and 7 clusters produced by HC. Overlap with clusters of the optimal solution of PAM with 7 clusters are listed in percent. Clusters are named according to the interpretation of the respective medoid sequence.

Abbreviations: PAM = partitioning-around-medoids, HC = hierarchical clustering, TF = treatment failure, AD = antidepressant‑monotherapy, MS = persistent anticonvulsant mood-stabilizers, AP = persistent antipsychotics, LI = persistent lithium, AUG = augmentation, LI AUG = lithium augmentation.

|  | TF n=8382  34.1% | AD-M n=4601  18.7% | Aug. n=1238  5.0% | Li Aug. n=1273  5.2% | AP n=2197  8.9% | Li n=2040  8.3% | MS n=4847  19.7% |
| --- | --- | --- | --- | --- | --- | --- | --- |
| Age Group | | |  |  |  |  |  |
| 16 - 35 | 5069 (60.5%) | 1998 (43.4%) | 602 (48.6%) | 664 (52.2%) | 1034 (47.1%) | 986 (48.3%) | 2667 (55.0%) |
| 36 - 65 | 3313 (39.5%) | 2603 (56.6%) | 636 (51.4%) | 609 (47.8%) | 1163 (52.9%) | 1054 (51.7%) | 2180 (45.0%) |
| Sex |  |  |  |  |  |  |  |
| Men | 3234 (38.6%) | 1445 (31.4%) | 448 (36.2%) | 500 (39.3%) | 890 (40.5%) | 848 (41.6%) | 1582 (32.6%) |
| Women | 5148 (61.4%) | 3156 (68.6%) | 790 (63.8%) | 773 (60.7%) | 1307 (59.5%) | 1192 (58.4%) | 3265 (67.4%) |
| Living with Partner | | | |  |  |  |  |
| No | 6487 (77.4%) | 3121 (67.8%) | 871 (70.4%) | 791 (62.1%) | 1559 (71.0%) | 1299 (63.7%) | 3296 (68.0%) |
| Yes | 1895 (22.6%) | 1480 (32.2%) | 367 (29.6%) | 482 (37.9%) | 638 (29.0%) | 741 (36.3%) | 1551 (32.0%) |
| Living with Children (<18) | | |  |  |  |  |  |
| No | 6188 (73.8%) | 3082 (67.0%) | 836 (67.5%) | 808 (63.5%) | 1591 (72.4%) | 1352 (66.3%) | 3228 (66.6%) |
| Yes | 2194 (26.2%) | 1519 (33.0%) | 402 (32.5%) | 465 (36.5%) | 606 (27.6%) | 688 (33.7%) | 1619 (33.4%) |
| Education | | |  |  |  |  |  |
| >12 years | 1936 (23.1%) | 1452 (31.6%) | 392 (31.7%) | 429 (33.7%) | 633 (28.8%) | 700 (34.3%) | 1585 (32.7%) |
| 0-9 years | 2442 (29.1%) | 892 (19.4%) | 247 (20.0%) | 246 (19.3%) | 492 (22.4%) | 370 (18.1%) | 900 (18.6%) |
| 10-12 years | 4004 (47.8%) | 2257 (49.1%) | 599 (48.4%) | 598 (47.0%) | 1072 (48.8%) | 970 (47.5%) | 2362 (48.7%) |
| Sickness Absence | | |  |  |  |  |  |
| >90 | 1405 (16.8%) | 1102 (24.0%) | 291 (23.5%) | 278 (21.8%) | 489 (22.3%) | 418 (20.5%) | 982 (20.3%) |
| 1-90 | 1107 (13.2%) | 789 (17.1%) | 232 (18.7%) | 261 (20.5%) | 361 (16.4%) | 374 (18.3%) | 807 (16.6%) |
| None | 5870 (70.0%) | 2710 (58.9%) | 715 (57.8%) | 734 (57.7%) | 1347 (61.3%) | 1248 (61.2%) | 3058 (63.1%) |
| Disability Pension | | |  |  |  |  |  |
| No | 7468 (89.1%) | 3789 (82.4%) | 1116 (90.1%) | 1181 (92.8%) | 1876 (85.4%) | 1865 (91.4%) | 4337 (89.5%) |
| Yes | 914 (10.9%) | 812 (17.6%) | 122 (9.9%) | 92 (7.2%) | 321 (14.6%) | 175 (8.6%) | 510 (10.5%) |
| History of Suicide Attempt | | |  |  |  |  |  |
| No | 7834 (93.5%) | 4285 (93.1%) | 1123 (90.7%) | 1183 (92.9%) | 2004 (91.2%) | 1926 (94.4%) | 4534 (93.5%) |
| Yes | 548 (6.5%) | 316 (6.9%) | 115 (9.3%) | 90 (7.1%) | 193 (8.8%) | 114 (5.6%) | 313 (6.5%) |
| Depression (F32-39) | |  |  |  |  |  |  |
| No | 5451 (65.0%) | 2457 (53.4%) | 687 (55.5%) | 737 (57.9%) | 1223 (55.7%) | 1223 (60.0%) | 2764 (57.0%) |
| Yes | 2931 (35.0%) | 2144 (46.6%) | 551 (44.5%) | 536 (42.1%) | 974 (44.3%) | 817 (40.0%) | 2083 (43.0%) |
| Substance Use (F10-19) | | | | | |  |  |
| No | 7279 (86.8%) | 4049 (88.0%) | 1036 (83.7%) | 1129 (88.7%) | 1846 (84.0%) | 1859 (91.1%) | 4343 (89.6%) |
| Yes | 1103 (13.2%) | 552 (12.0%) | 202 (16.3%) | 144 (11.3%) | 351 (16.0%) | 181 (8.9%) | 504 (10.4%) |
| Neurotic Disorder (F40-49) | | |  |  |  |  |  |
| No | 5663 (67.6%) | 2705 (58.8%) | 721 (58.2%) | 869 (68.3%) | 1310 (59.6%) | 1473 (72.2%) | 3262 (67.3%) |
| Yes | 2719 (32.4%) | 1896 (41.2%) | 517 (41.8%) | 404 (31.7%) | 887 (40.4%) | 567 (27.8%) | 1585 (32.7%) |
| Behavioral Disorder (F50-59) | | |  |  |  |  |  |
| No | 7994 (95.4%) | 4358 (94.7%) | 1176 (95.0%) | 1208 (94.9%) | 2099 (95.5%) | 1976 (96.9%) | 4625 (95.4%) |
| Yes | 388 (4.6%) | 243 (5.3%) | 62 (5.0%) | 65 (5.1%) | 98 (4.5%) | 64 (3.1%) | 222 (4.6%) |
| Personality Disorder (F60-69) | | | |  |  |  |  |
| No | 7874 (93.9%) | 4270 (92.8%) | 1167 (94.3%) | 1216 (95.5%) | 2044 (93.0%) | 1950 (95.6%) | 4568 (94.2%) |
| Yes | 508 (6.1%) | 331 (7.2%) | 71 (5.7%) | 57 (4.5%) | 153 (7.0%) | 90 (4.4%) | 279 (5.8%) |
| ADHD (F90) | | |  |  |  |  |  |
| No | 7649 (91.3%) | 4243 (92.2%) | 1152 (93.1%) | 1217 (95.6%) | 2027 (92.3%) | 1950 (95.6%) | 4518 (93.2%) |
| Yes | 733 (8.7%) | 358 (7.8%) | 86 (6.9%) | 56 (4.4%) | 170 (7.7%) | 90 (4.4%) | 329 (6.8%) |
| Cancer |  |  |  |  |  |  |  |
| No | 7892 (94.2%) | 4232 (92.0%) | 1167 (94.3%) | 1195 (93.9%) | 2041 (92.9%) | 1940 (95.1%) | 4540 (93.7%) |
| Yes | 490 (5.8%) | 369 (8.0%) | 71 (5.7%) | 78 (6.1%) | 156 (7.1%) | 100 (4.9%) | 307 (6.3%) |
| Obesity/Diabetes | | |  |  |  |  |  |
| No | 8202 (97.9%) | 4428 (96.2%) | 1190 (96.1%) | 1244 (97.7%) | 2088 (95.0%) | 1991 (97.6%) | 4673 (96.4%) |
| Yes | 180 (2.1%) | 173 (3.8%) | 48 (3.9%) | 29 (2.3%) | 109 (5.0%) | 49 (2.4%) | 174 (3.6%) |
| Neurolgical Disorder | | |  |  |  |  |  |
| No | 7787 (92.9%) | 4179 (90.8%) | 1133 (91.5%) | 1178 (92.5%) | 1988 (90.5%) | 1900 (93.1%) | 4464 (92.1%) |
| Yes | 595 (7.1%) | 422 (9.2%) | 105 (8.5%) | 95 (7.5%) | 209 (9.5%) | 140 (6.9%) | 383 (7.9%) |
| Muscolosceletal Disorder | | |  |  |  |  |  |
| No | 7104 (84.8%) | 3793 (82.4%) | 1038 (83.8%) | 1095 (86.0%) | 1809 (82.3%) | 1756 (86.1%) | 4082 (84.2%) |
| Yes | 1278 (15.2%) | 808 (17.6%) | 200 (16.2%) | 178 (14.0%) | 388 (17.7%) | 284 (13.9%) | 765 (15.8%) |
| Cardiorespiratory Disorder | | |  |  |  |  |  |
| No | 7915 (94.4%) | 4223 (91.8%) | 1150 (92.9%) | 1206 (94.7%) | 2018 (91.9%) | 1926 (94.4%) | 4557 (94.0%) |
| Yes | 467 (5.6%) | 378 (8.2%) | 88 (7.1%) | 67 (5.3%) | 179 (8.1%) | 114 (5.6%) | 290 (6.0%) |

**Supplementary Table 8.** Distribution of sociodemographic variables by sequence typology.

Abbreviations: TF = Treatment Failure, AD-M = Persistent Antidepressant-Monotherapy, MS = Persistent Mood-Stabilizers, Li = Persistent Lithium, AP = Persistent Antipsychotics, Aug. = Persistent Augmentation, Li Aug. = Persistent Augmentation with Lithium

| **Cluster** | **Swedish-born** | **2nd-generation immigrants** | **Non-refugee immigrants** | **Refugees** |
| --- | --- | --- | --- | --- |
| ***Treatment failure*** | 6586 (78.6%) | 762  (9.1%) | 605  (7.2%) | 429  (5.1%) |
| (Reference) | / | / | / | / |
| ***Peristent antidepressant monotherapy*** | 3851 (83.7%) | 309  (6.7%) | 275  (6.0%) | 166  (3.6%) |
| Raw OR (CI) |  | 0.7 (0.6-0.8) *** | 0.8 (0.7-0.9) ** | 0.7 (0.6-0.8) *** |
| Adjusted OR (CI) |  | 0.8 (0.7-0.9) ** | 0.7 (0.5-0.8) *** | 0.5 (0.4-0.7) *** |
| ***Peristent Mood-stabilizers*** | 4256 (87.8%) | 306  (6.3%) | 205  (4.2%) | 80  (1.7%) |
| Raw OR (CI) |  | 0.6 (0.5-0.7) *** | 0.5 (0.4-0.6) *** | 0.3 (0.2-0.4) *** |
| Adjusted OR (CI) |  | 0.7 (0.6-0.8) *** | 0.5 (0.4-0.7) *** | 0.3 (0.2-0.4) *** |
| ***Peristent Antipsychotics*** | 1838 (83.7%) | 147  (6.7%) | 131  (6.0%) | 81  (3.7%) |
| Raw OR (CI) |  | 0.7 (0.6-0.8) *** | 0.8 (0.6-0.9) * | 0.7 (0.5-0.9) * |
| Adjusted OR (CI) |  | 0.8 (0.6-0.9) * | 0.7 (0.5-0.9) * | 0.6 (0.4-0.8) * |
| ***Peristent Lithium*** | 1725 (84.6%) | 149  (7.3%) | 116  (5.7%) | 50  (2.5%) |
| Raw OR (CI) |  | 0.7 (0.6-0.9) * | 0.7 (0.6-0.9) * | 0.4 (0.3-0.6) *** |
| Adjusted OR (CI) |  | 0.8 (0.7-1.0) * | 0.7 (0.5-0.9) * | 0.4 (0.3-0.7) *** |
| ***Peristent Augmentation*** | 1053 (85.1%) | 92  (7.4%) | 60  (4.8%) | 33  (2.7%) |
| Raw OR (CI) |  | 0.8 (0.6-0.9) * | 0.6 (0.5-0.8) ** | 0.5 (0.3-0.7) *** |
| Adjusted OR (CI) |  | 0.8 (0.7-1.0) | 0.6 (0.4-0.9) * | 0.5 (0.3-0.8) * |
| ***Peristent Augmentation***  ***with Lithium*** | 1052 (82.6%) | 102  (8.0%) | 70  (5.5%) | 49  (3.8%) |
| Raw OR (CI) |  | 0.8 (0.7-1.0) | 0.7 (0.6-0.9) * | 0.7 (0.5-0.9) * |
| Adjusted OR (CI) |  | 0.9 (0.7-1.1) | 0.6 (0.4-0.8) * | 0.5 (0.3-0.8) * |

**Supplementary Table 9.** Multinomial regression results, specifying odds ratios (OR) with confidence intervals (CI) for treatment typologies respective of population group. OR are relative to the majority treatment typology of treatment failure and the majority population of Swedish-born subjects. * indicated p < 0.05, ** p < 0.01 and *** p < 0.001.


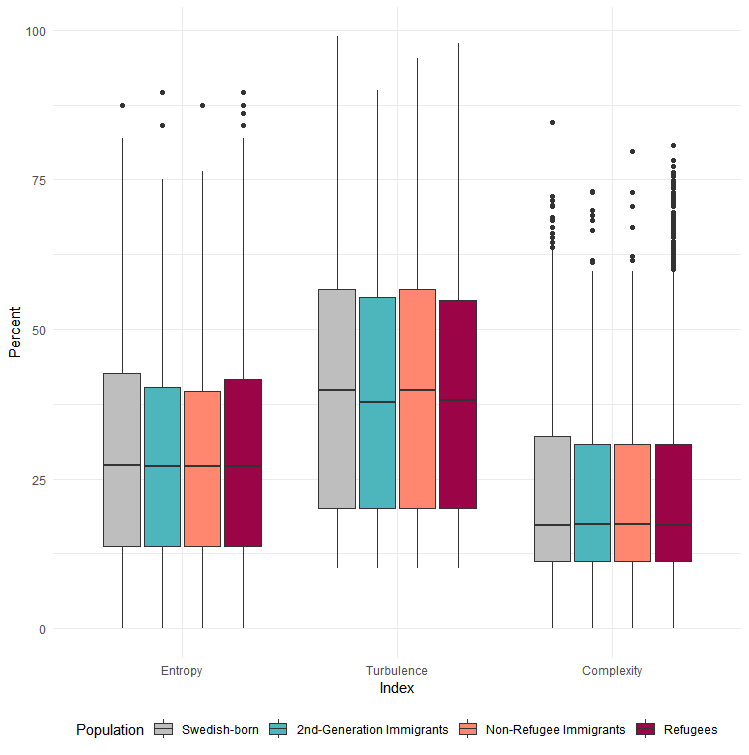


**Supplementary Figure 1.** Boxplot of longitudinal sequence entropy (number of observed states relative to the maximum possible number of states), turbulence and complexity (composite measures of observed states and transitions). No significant differences in mean values were observed between the four population-groups.

**
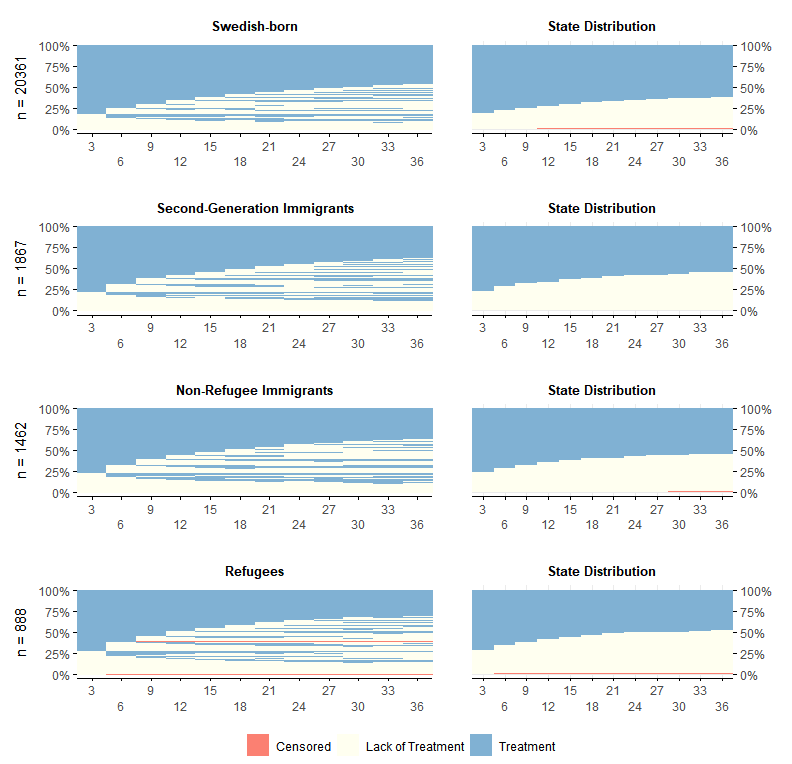
**

**Supplementary Figure 2.** Dichotomized sequence analysis results, contrasting any treatment states to lack of treatment and censored states. Observed sequences and yearly state distribution, respectively for population-groups.

.


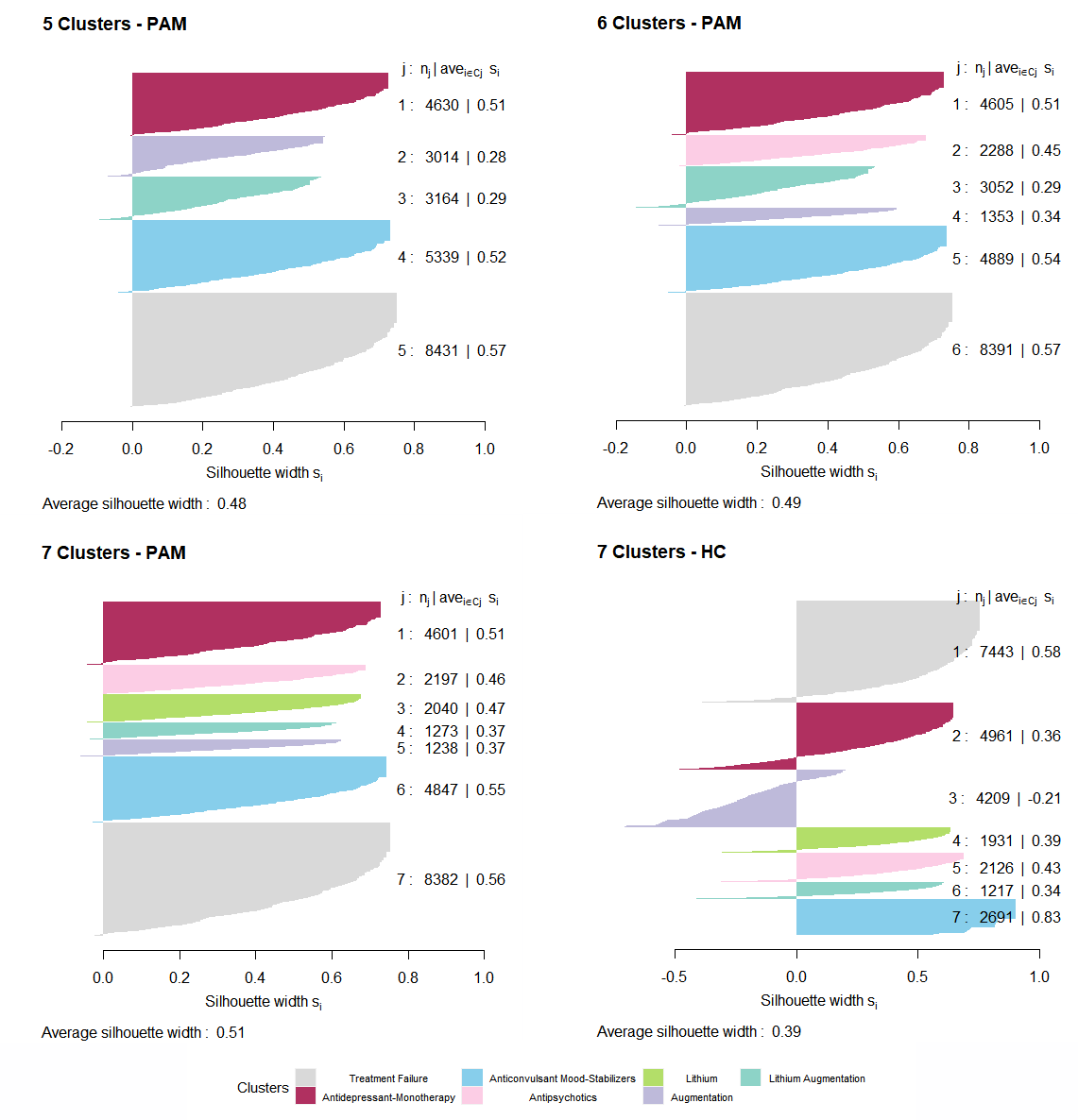


**Supplementary Figure 3.** Silhouette plots of average silhouette width for the total cohort as well as each cluster produced respectively by 5-, 6- and 7-object solutions by the partitioning around-medoids (PAM) algorithm, and the 7-object solution produced by hierarchical clustering (HC). Clusters are colored and named by respective medoid sequences.

**
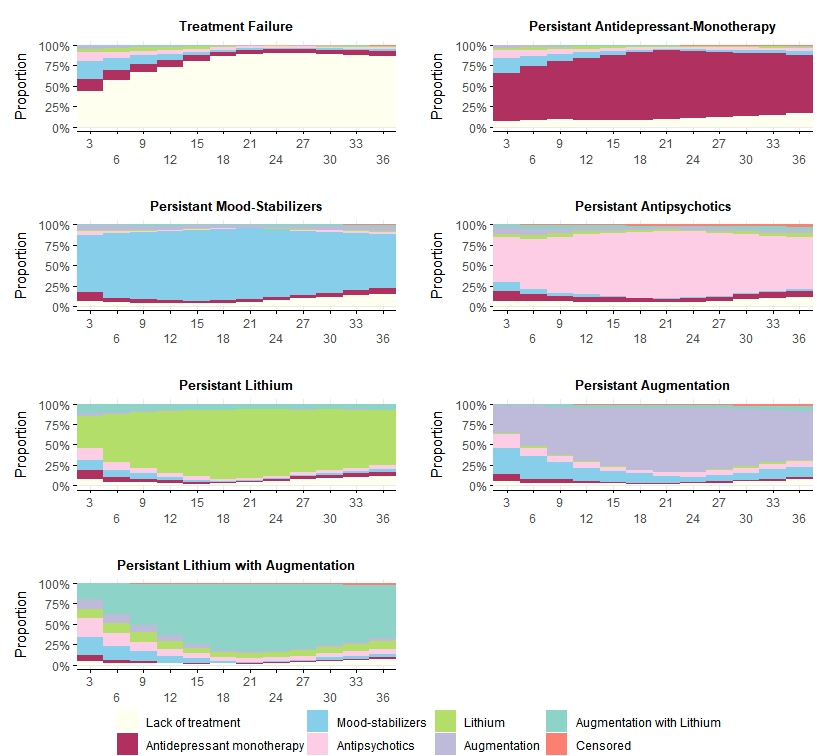
**

**Supplementary Figure 4.** State distribution across the follow-up of 3 years, displayed as 12 periods of 91 days, observed respectively among the seven cluster typologies

**
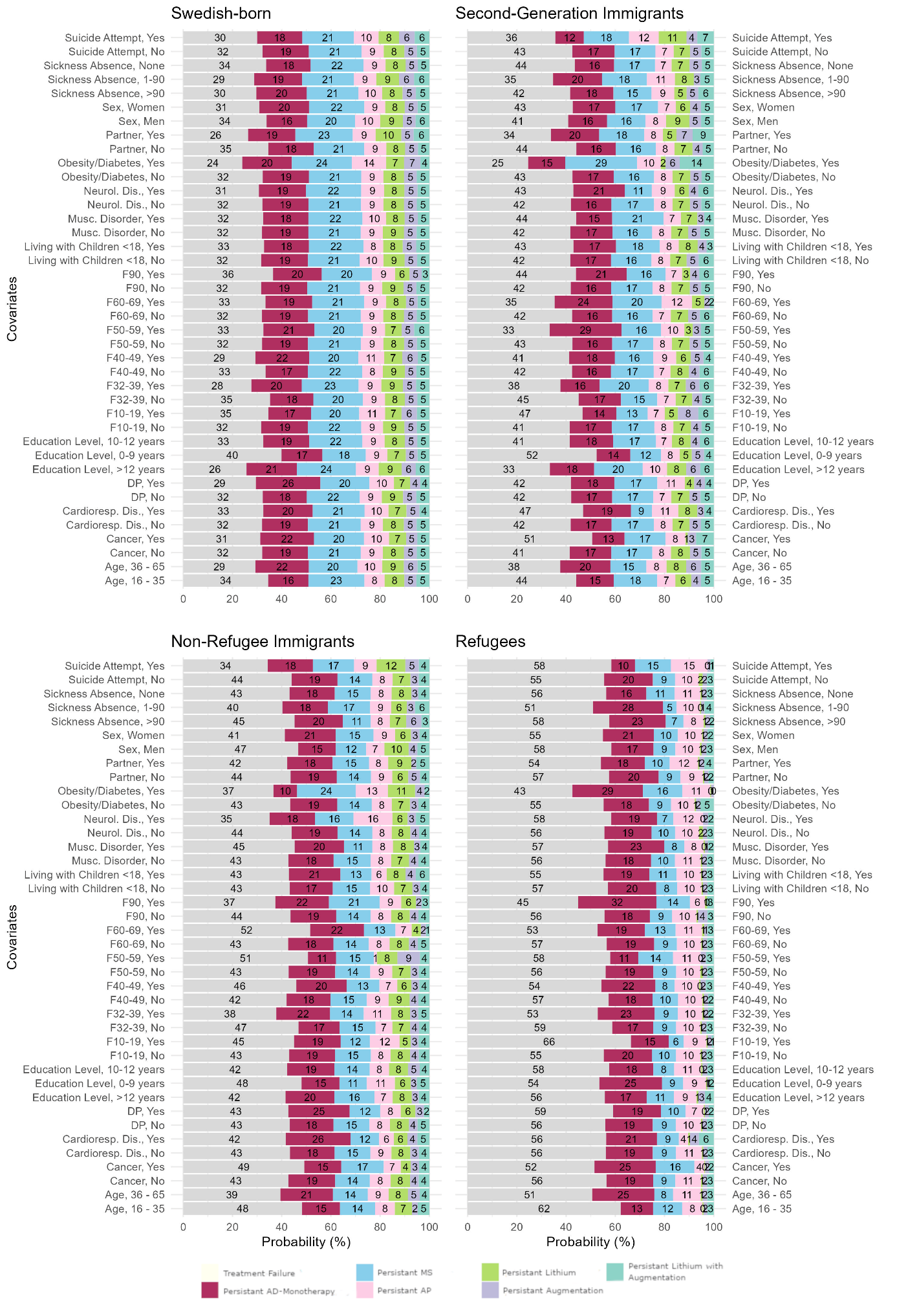
**

**Supplementary Figure 5.** Multinomial regression results displayed as probabilities of cluster membership. Estimates are provided for each predictor factor level adjusted for the remainder of covariates, respectively among A) Swedish-born, B) 2^nd^ generation immigrants, C) non‑refugee 1^st^ generation immigrants, and D) refugees.

**References**

**Brooke, H. L., Talback, M., Hornblad, J., Johansson, L. A., Ludvigsson, J. F., Druid, H., Feychting, M. & Ljung, R.** (2017). The Swedish cause of death register. *Eur J Epidemiol* **32**, 765-773.

**Ekbom, A.** (2011). The Swedish Multi-generation Register. *Methods Mol Biol* **675**, 215-20.

**Försäkringskassan** (2018). Försäkringskassans mikrodata över socialförsäkringen. [**https://www.registerforskning.se/wp-content/uploads/2018/10/OlofHakansson_Fk.pdf**](https://www.registerforskning.se/wp-content/uploads/2018/10/OlofHakansson_Fk.pdf).

**Ludvigsson, J. F., Almqvist, C., Bonamy, A. K., Ljung, R., Michaelsson, K., Neovius, M., Stephansson, O. & Ye, W.** (2016). Registers of the Swedish total population and their use in medical research. *Eur J Epidemiol* **31**, 125-36.

**Ludvigsson, J. F., Andersson, E., Ekbom, A., Feychting, M., Kim, J. L., Reuterwall, C., Heurgren, M. & Olausson, P. O.** (2011). External review and validation of the Swedish national inpatient register. *BMC Public Health* **11**, 450.

**Ludvigsson, J. F., Svedberg, P., Olén, O., Bruze, G. & Neovius, M.** (2019). The longitudinal integrated database for health insurance and labour market studies (LISA) and its use in medical research. *European Journal of Epidemiology* **34**, 423-437.

**Statistics Sweden** (2018). STATIV - a longitudinal database for integration studies. [**https://www.scb.se/contentassets/659b9a5233dc4dd49b22630b2745ca57/informationsfolder-stativ-eng_mars2018.pdf**](https://www.scb.se/contentassets/659b9a5233dc4dd49b22630b2745ca57/informationsfolder-stativ-eng_mars2018.pdf).

**Wettermark, B., Hammar, N., Fored, C. M., Leimanis, A., Otterblad Olausson, P., Bergman, U., Persson, I., Sundstrom, A., Westerholm, B. & Rosen, M.** (2007). The new Swedish Prescribed Drug Register--opportunities for pharmacoepidemiological research and experience from the first six months. *Pharmacoepidemiol Drug Saf* **16**, 726-35.
